# Repression of Fyn-related kinase in breast cancer cells is associated with promoter site-specific CpG methylation

**DOI:** 10.18632/oncotarget.14546

**Published:** 2017-01-06

**Authors:** Edward T. Bagu, Sayem Miah, Chenlu Dai, Travis Spriggs, Yetunde Ogunbolude, Erika Beaton, Michelle Sanders, Raghuveera K. Goel, Keith Bonham, Kiven E. Lukong

**Affiliations:** ^1^ Department of Biochemistry, College of Medicine, University of Saskatchewan, Saskatoon, Saskatchewan, S7N 5E5, Canada; ^2^ Cancer Research Unit, Health Research Division, Saskatchewan Cancer Agency, and Division of Oncology, College of Medicine, University of Saskatchewan, Saskatoon, SK S7N 4H4, Canada; ^3^ Stowers Institute for Medical Research, Kansas City, MO 64110, USA

**Keywords:** fyn-related kinase, FRK, BRK, breast cancer, triple negative breast cancer

## Abstract

The triple-negative breast cancer subtype is highly aggressive and has no defined therapeutic target. Fyn-related kinase (FRK) is a non-receptor tyrosine kinase, reported to be downregulated in breast cancer and gliomas, where it is suggested to have tumor suppressor activity. We examined the expression profile of FRK in a panel of 40 breast cancer cells representing all the major subtypes, as well as in 4 non-malignant mammary epithelial cell lines. We found that FRK expression was significantly repressed in a proportion of basal B breast cancer cell lines. We then determined the mechanism of suppression of FRK in FRK-low or negative cell lines. *In silico* analyses of the FRK promoter region led to the identification of at least 17 CpG sites. Bisulphite sequencing of the promoter region revealed that two of these sites were consistently methylated in FRK-low/negative cell lines and especially in the basal B breast cancer subtype. We further show that treatment of these cells with histone deacetylase inhibitors, Entinostat and Mocetinostat' promoted re-expression of FRK mRNA and protein. Further, using luciferase reporter assays, we show that both GATA3-binding protein FOG1 and constitutively active STAT5A increased the activity of FRK promoter. Together, our results present the first evidence that site-specific promoter methylation contributes to the repression of *FRK* more so in basal B breast cancers. Our study also highlights the potential clinical significance of targeting FRK using epigenetic drugs specifically in basal B breast cancers which are usually triple negative and very aggressive.

## INTRODUCTION

Breast cancer is the most common form of cancer affecting women worldwide, with 1.7 million new cases diagnosed each year [1]. Gene expression profiling has enabled the classification of breast cancer into four subtypes: human epithelial growth factor receptor 2-positive, luminal A and B, and basal or the triple-negative breast cancer [2]. Basal breast cancers cell lines have been subdivided into A and B sub-categories [3, 4]. Basal A cell lines are associated with the up-regulation of several genes in the E-twenty six transformation-specific pathway (ETS) and mutations of the tumor suppressor genes BRCA1 and 2; while, basal B cell lines are claudin-low and display mesenchymal and stem cell-like characteristics [3, 4].

Fyn-Related Kinase (FRK) is a non-receptor tyrosine kinase coded by *FRK* located on chromosome 6q21–23, a region that displays loss of heterozygosity (LOH) in nearly 30% of breast cancers [5, 6]. FRK belongs to the breast tumor kinase (BRK) family kinases (BFKs) that includes BRK and SRMS [7, 8]. BFKs share a conserved intron-exon architecture distinct from that of their closest relatives, the Src family kinases (SFKs) [7, 9]. Like SFKs, FRK is functionally composed of 3 domains, Src homology 3 (SH3), SH2 and a kinase domain. FRK possesses an auto-regulatory tyrosine residue (Y387) within the activation loop of the kinase domain and a putative C-terminal regulatory tyrosine (Y497) that is conserved in SFKs [10, 11].

There is evidence that FRK functions as a tumor suppressor [7, 12]. Knocking down *FRK* in the immortalized non-tumorigenic mammary epithelial cell line, MCF10A, induced transformation [13, 14]; while, exogenous expression of FRK in breast and brain cancer cells inhibited cell proliferation, migration and invasiveness [13, 15, 16]. FRK regulates cell growth by interacting with and/or phosphorylating specific cellular proteins [12, 14, 15, 17]. FRK was shown to interact with retinoblastoma protein (pRB), a tumor repressor gene, via the A/B pocket, inhibiting the proliferation of breast cancer cells [18]. Over-expression of FRK in glioblastoma cells downregulated phosphorylated pRB, leading to growth arrest in the G1-phase [19]. FRK was later shown to inhibit cell proliferation, invasion and colony formation in breast cancer cells devoid of pRB by the phosphorylation and stabilization of tumor suppressor PTEN [13]. Interestingly, the depletion of *Frk* expression in mice had no effect on tumor formation [6]. There are suggestions that FRK may be oncogenic in some cancers [12].

Previous analyses of FRK in breast cancer cells/tissues reported differential expression patterns [9, 20]. FRK was reported to be repressed in a panel of 21 invasive breast carcinoma tissues and in 20% of invasive ductal carcinoma tissues [21, 22]. Pajer*et al*. reported that provirus–induced insertional mutations in the *frk* promoter increased *frk* expression in chicken lung sarcomas [23].

At present, the mechanisms regulating the expression of FRK in breast cancer are unknown. Epigenetic alterations in tumor suppressor genes have been identified in breast and other forms of cancer [24, 25]. Aberrant promoter hypermethylation is a frequent event in the silencing of several tumor suppressor genes including BRCA1 and spleen tyrosine kinase in various cancers [26–30]. In this study, we investigated the expression of FRK and its promoter methylation status in breast cancer cell lines. We found that the *FRK* promoter is methylated at specific CpG sites in FRK-low/negative breast cancer cell lines and demonstrated that histone deacetylase inhibitors reactivated the expression of *FRK* in these cells.

## RESULTS

### FRK levels are repressed in a subset of human breast cancer cells

**Figure 1:**
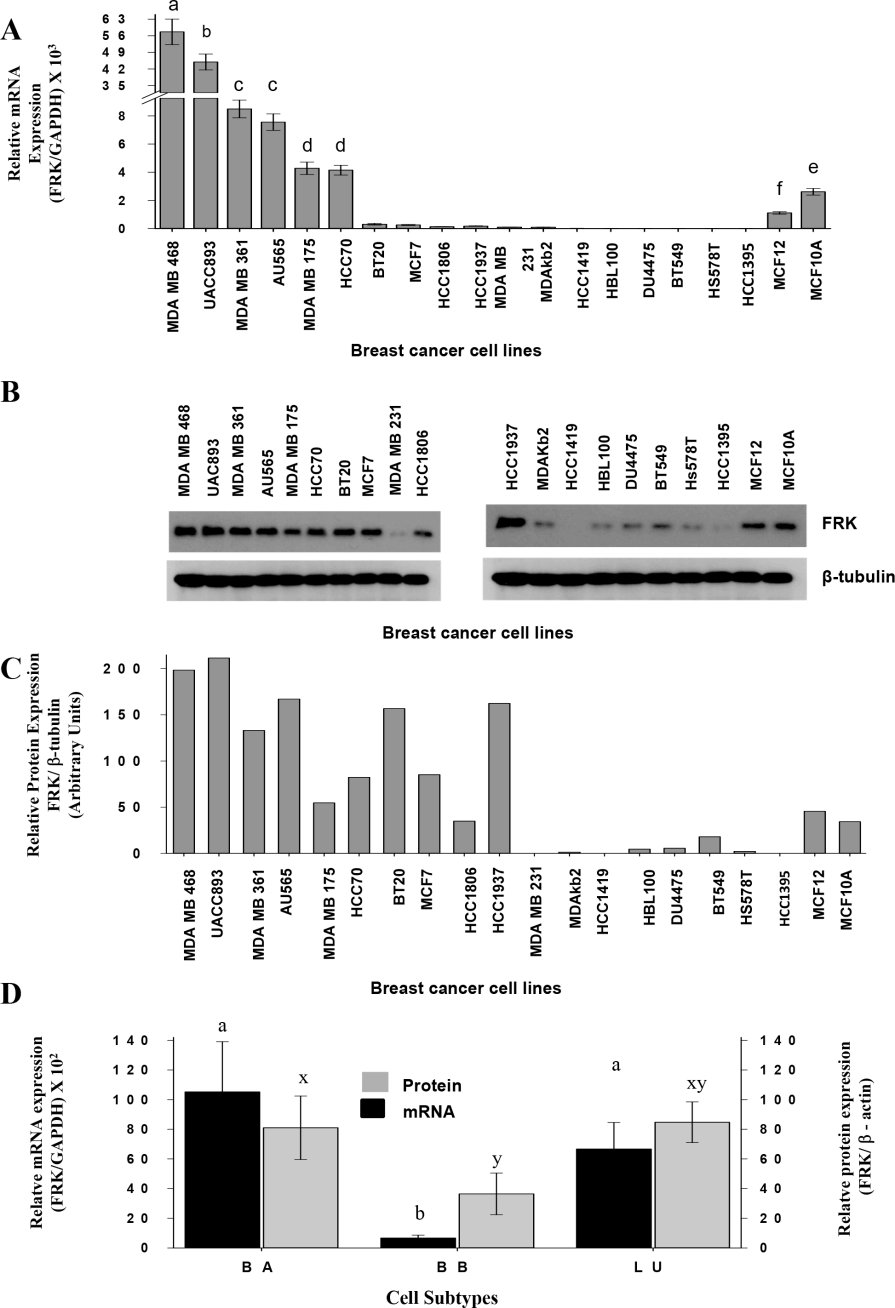
FRK levels are repressed in a subset of human breast cancer cells (**A**) *FRK* transcript levels relative to that of *GAPDH* in each breast cancer cell line was assessed by qRT-PCR and then normalized to that of the HCC1395 breast cancer cells to determine the relative FRK transcript abundance. (**B**) FRK protein levels in each cell was analyzed from the cell lysates by immunoblotting using an FRK antibody (Santa Cruz), β-tubulin, a house keeping gene was used as a loading control. (**C**) Mean protein expression levels from (B) were quantified by densitometry and presented as the relative FRK/ β-tubulin expression using arbitrary units. (**D**) The mean relative expression of FRK (mRNA and protein) and β-actin levels in 37 breast cancer cell lines that were classified in the three cohorts Luminal (LU), Basal A (BA) and Basal B (BB) breast cancer cells. The mean relative protein expression levels were quantified by densitometry analysis of immunoblots in the Supplementary Figure 1A. The FRK expression was determined relative to β-ACTIN, a house keeping gene. Data is presented as Mean ± SEM, different superscripts a-z are used to indicate significant differences across means (a-z = *P* ≤ 0.05).

Previous work produced conflicting data regarding the expression of FRK in human breast cancers and cell lines [9, 31–33]. To clarify this, we examined the expression of FRK in 44 cell lines. In Figure 1A to 1C, we present results for 20 cell lines with the highest and lowest FRK expression. Most of the low FRK expressing breast cell lines were the basal B cell lines (MDA-MB-231; HBL100; BT549; Hs578T; HCC1395), some luminal (MDA-kb2, HCC1419) and basal A (DU4475) cells had low levels (Figure 1A to 1C). Based on the densitometry analysis of immunoblots of 37 cell lines (Supplementary Figure 1A), mean FRK levels were lower in the basal B as compared to either luminal or basal A cell lines (*P* < 0.05; Figure 1D). *FRK* transcript levels were correlated with protein levels (*n* = 37, R = 0.63; *P* < 0.05). Loss of FRK expression is more prevalent in the basal B breast cancers than other subtypes. Taken together, our data indicate that FRK is differentially expressed in breast cancer and the loss of FRK expression is more prevalent in the basal B breast cancers than other subtypes.

### The expression of *FRK* in breast cancer cells correlates with site-specific promoter methylation

**Figure 2:**
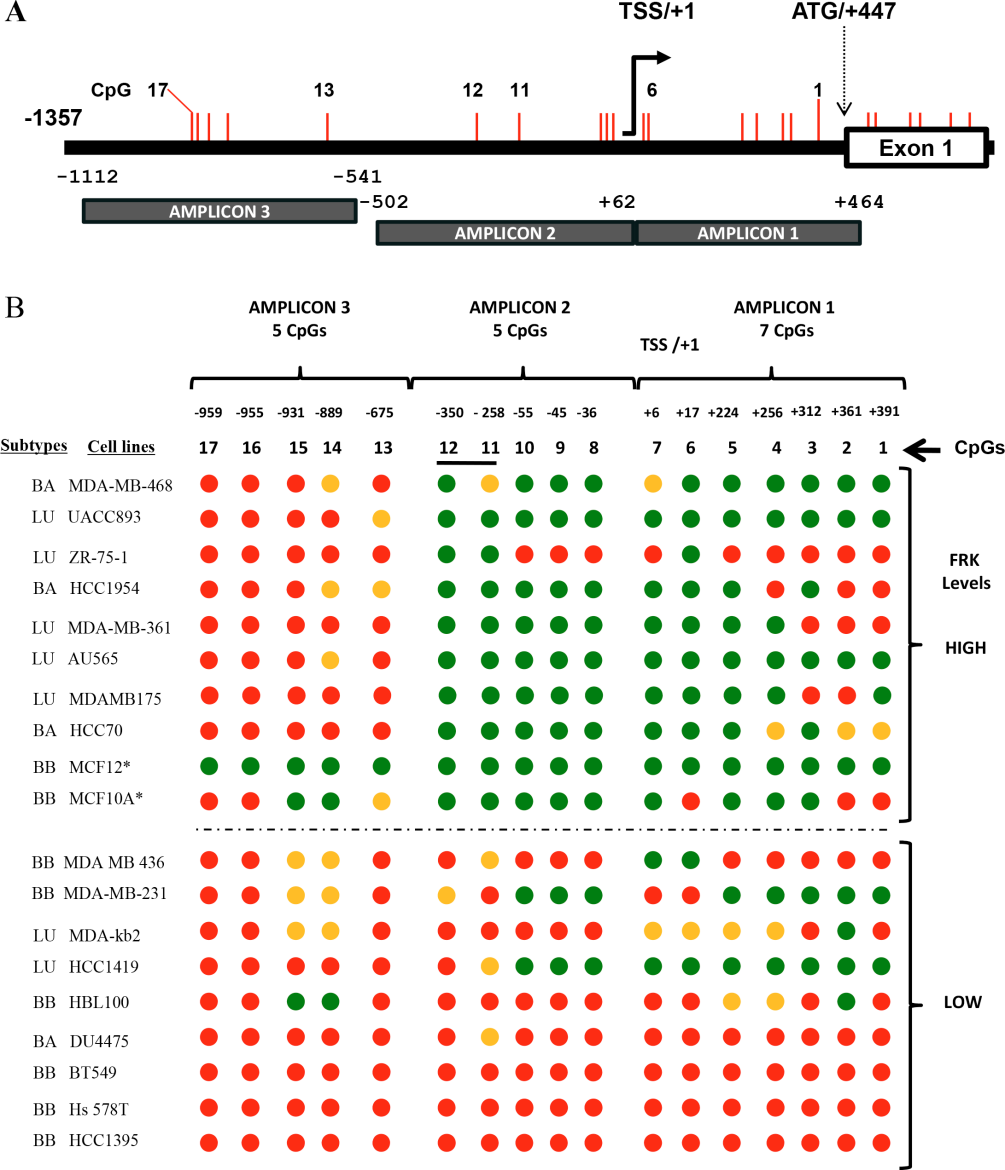
The expression of *FRK* in breast cancer cells correlates with site-specific promoter methylation (**A**) A schematic representation of the FRK promoter region, showing CpG sites as vertical red lines. Methylation specific primers were designed spanning the 2 regions, +464/−502 and −541/−1112 of the 5′ un-translated region (UTR) and the non-coding region up-stream up stream of exon 1 (ATG/+447), using a bioinformatics tool 65. (**B**) The methylation status of 17 CpG sites, numbered 1 to 17, from +391 to -959 bp of the transcriptional start site (TSS /+1) was determined. Genomic DNA was extracted from breast cancer cell lines or cell lines derive from normal epithelium (MCF10A and MCF12) with either low or high FRK mRNA expression and treated with sodium bisulfite, the DNA sequence of each amplicon was then determined to evaluate the methylation status of each of the 17 CpGs in the FRK promoter region numbered from +391 to -959 of the TSS/+1. Red, green and orange circles represented the methylated, non-methylated and differentially methylated CpG sites (hemi-methylated), respectively. Asterisks (*) refers to the cell lines MCF10A and MCF12 that are derived from normal human breast epithelium.

FRK is a candidate tumor suppressor [15, 17, 34]. Aberrant methylation of CpG sites in the promoter region of tumor repressor genes is frequently observed in most cancers [35–37]. We therefore hypothesized that DNA methylation decreased the expression of *FRK* in a subset of breast cancer cells. We analyzed the DNA sequence of the *FRK* promoter (+447/−1357) using bisulphite primers that span regions, +464/−502 and −541/−1112 relative to the transcriptional start site (TSS/+1; Figure 2A). The *FRK* promoter is devoid of a classic CpG island; however, there are 17 CpGs, from position +391 to -959 bp, which we numbered accordingly (Figure 2B). In Figure 2B, we show 19 of the 34 breast cancer cell lines with the highest (*n* = 8) and lowest (*n* = 9) FRK expression levels. MCF10A and MCF12 are non-transformed mammary epithelial cell lines and both displayed varied methylation status. The FRK promoter region, +391 to -350, was extensively methylated in cells that expressed low as compared to high FRK levels, with exception of the luminal breast cancer cell lines, HCC1419 and ZR-75-1 (Figure 2B). In the latter two cell lines the reverse methylation patterns were seen (Figure 2B). Two CpGs, 11 and 12 at sites -258 and -350 respectively, of *FRK* promoter were consistently methylated in cells expressing low as compared to high FRK levels (Figure 2B). In the 36 cell lines analyzed, the incidence of methylation at CpGs, 11 and 12, sites -258 and -350 respectively was 27.8 %. Interestingly, CpG 11 at site -258 from the TSS/+1 was hemi-methylated in 16.7 % of the cell analyzed. CpGs in the distal promoter region (−675/−959) were methylated in all the breast cancer cells except the basal B, immortalized non-transformed cell lines MCF12 (Figure 2B). Overall, our data indicate that CpGs 11 and 12, at sites -258 and -350 are crucial for the expression of *FRK*, and suggest the FRK promoter methylation is site-specific.

### Decitabine induces the expression of *FRK* in breast cancer cells

**Figure 3:**
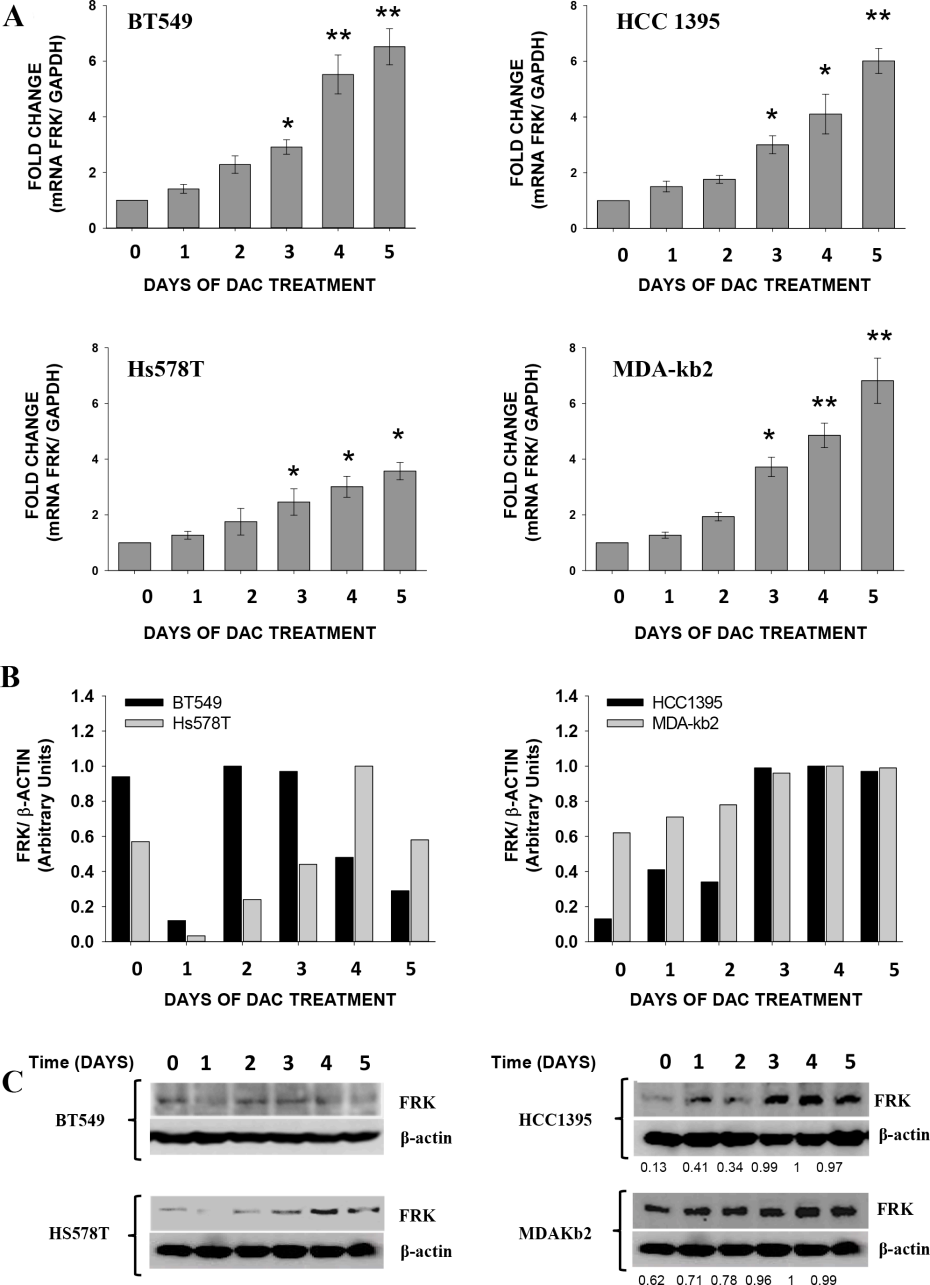
Decitabine (DAC) induces the expression of *FRK* in breast cancer cells Breast cancer cells were treated with either 5 μM (Hs578T cells) or 10 μM (HCC1395 and MDA-Kb2 cells) of DAC every day for different durations indicated on the x-axis. (**A**) FRK mRNA was extracted and quantified by qRT-PCR, FRK expression was determined relative to the GAPDH, then the fold change calculated relative to the controls. Data is presented as Mean ± SEM, asterisks represent mean values greater than controls (*P ≤ 0.05 and **P ≤ 0.01). (**B**) Densitometry analysis of the immunoblots of the FRK protein levels. (**C**) The FRK protein levels in each cell lysate was analyzed by immunoblotting using an FRK antibody. The mean relative protein expression levels were quantified by densitometry analysis of immunoblots. The FRK expression was determined relative to β-ACTIN, a house keeping gene and in each cell line normalized with the highest density ratio in each blot (1).

Decitabine (DAC) was shown to re-activate the expression of silenced genes in tumour cells by passive inhibition of DNA methyltransferase (DNMT) 1 [35, 37, 38]. We therefore examined if DAC would reactivate the expression of *FRK* in 4 breast cancer cell lines (BT549, HCC1395, Hs578T and MDA-kb2) with extensive *FRK* promoter methylation and low FRK expression. In all cases *FRK* expression was increased following treatment with DAC (*P* < 0.05; Figure 3A). FRK protein levels were also elevated in HCC1395, Hs578T and MDA-kb2 cells (Figure 3B and 3C). Our data therefore demonstrates that DAC induces the expression *FRK* in cells with low FRK expression levels and extensive promoter CpG-methylation and our findings are consistent with the notion that the methylation status of the *FRK* promoter is essential for gene expression.

### HDAC inhibitors increase the expression of FRK in breast cancer cells

**Figure 4:**
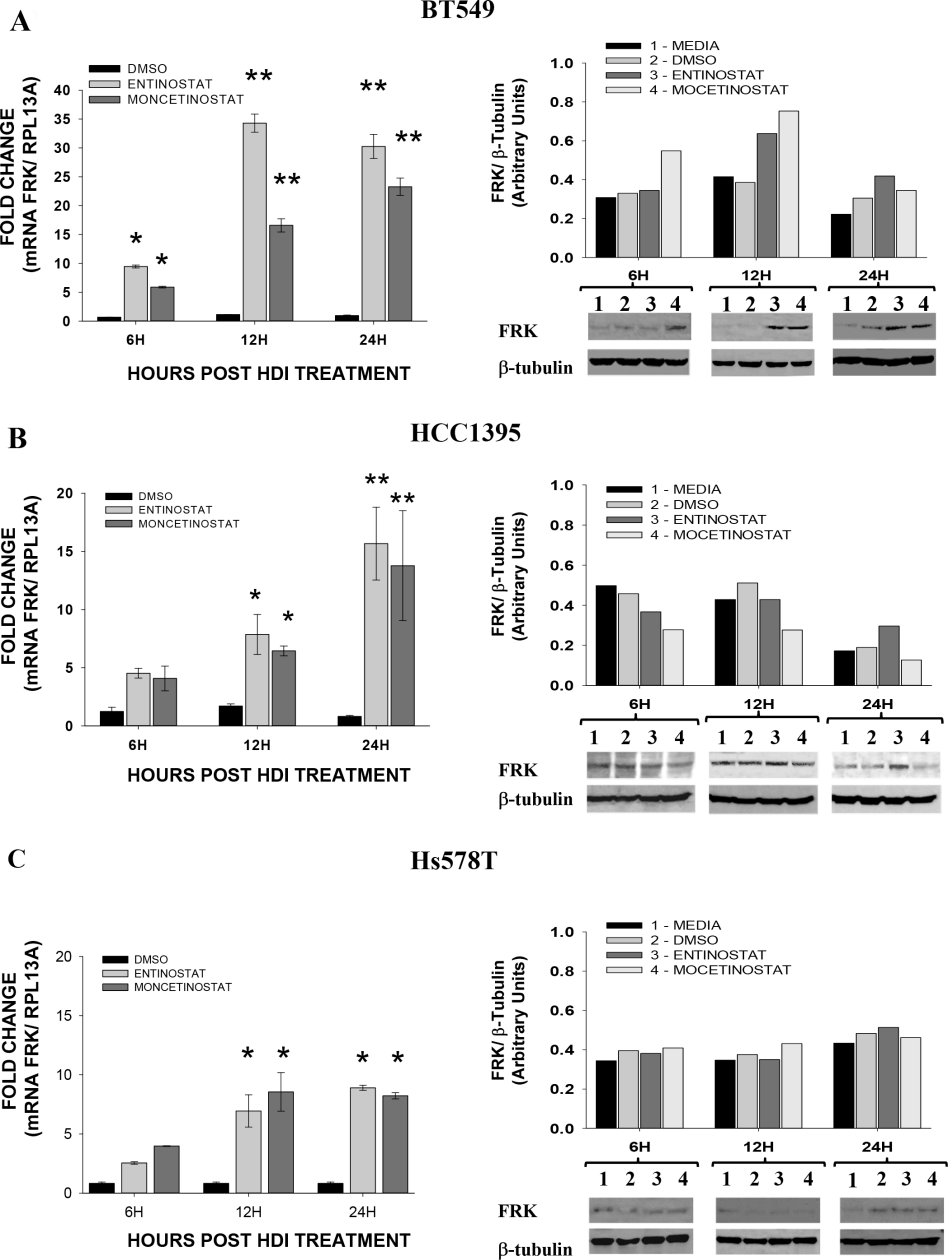
Histone deacetylase Inhibitors induces the expression of *FRK* in breast cancer cells Breast cancer cellsBT549 (**A**), HCC1395 (**B**) and Hs578T (**C**) were treated with either Entinostat (MS275; 2 μM) or Mocetinostat (MGCD0103; 1 μM) dissolved in DMSO, for the time periods of 6, 12 and 24, hours, indicated on x-axis as 6H, 12H, and 24H respectively; while, controls at all the given time points received the vehicle (DMSO; 0.2 μl/ ml). To evaluate the relative FRK transcript abundance post-treatment, the FRK transcript levels in cells were determined relative to that of the house keeping gene, (Ribosomal Protein L13a) RPL13A using qRT-PCR. The fold change was then calculated relative to the controls in each experiment. Data is presented as Mean ± SEM, asterisks represent mean values greater than controls (*P ≤ 0.05 and **P ≤ 0.01). The FRK protein levels in each cell lysate was analyzed by immunoblotting using an FRK antibody, the mean relative protein expression levels were then quantified by densitometry analysis of immunoblots. The FRK expression was determined relative to β-TUBULIN, a house keeping gene and presented as arbitrary units.

Histone Deacetylase Inhibitors (HDIs) have been shown to activate silenced genes including *CDKN1A*, *CDKN2A,SALL3*, *RARb2, TERT* and *GATA4*, in the human cancer cells by active DNA demethylation of their respective promoters [39–41]. To determine whether the methylation status of FRK can be reversed by exposure to HDIs, we examined the effect of HDIs, Mocetinostat and Entinostat, on BT549, HCC1395, Hs578T and MDA-kb2 cell lines with extensive promoter methylation and low FRK expression levels. In all cases, the HDIs increased *FRK* levels 12 to 24 hours post-treatment (*P* < 0.05; Figure 4). Although an increase in FRK protein was only seen in the BT549 cells, at 12 and 24 hours post-treatment (*P* ≤ 0.05; Figure 4), our data as a whole demonstrates that both Mocetinostat and Entinostat effectively relieved the epigenetic silencing of *FRK* in cells with extensive promoter CpG-methylation and low FRK expression levels.

### Epigenetic drug alters the CpG methylation status of the FRK promoter

**Figure 5:**
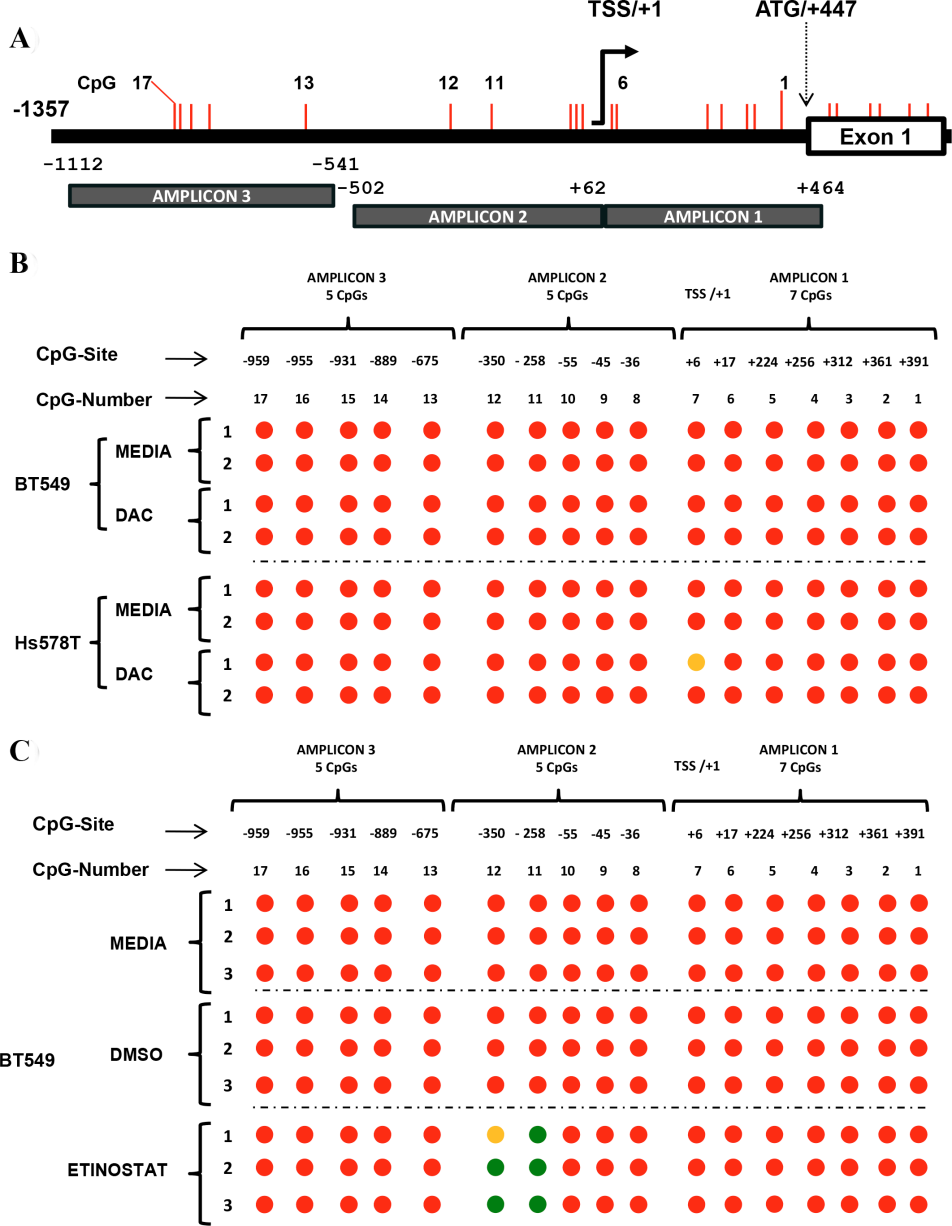
Epigenetic drugs alter the CpG methylation status of the FRK promoter (**A**) A schematic representation of the FRK promoter region up-stream of exon 1, showing the 17 CpG sites from the translation start site (ATG) at site +447. Methylation specific primers were designed spanning the 2 regions, +464/−502 and −541/−1112 of the 5′ un-translated region (UTR) and the non-coding region up-stream up stream of exon 1 (ATG/+447), using a bioinformatics tool [65]. The CpG sites are represented as vertical red lines and numbered 1 to 17, from +391 to −959 of the TSS/+1, respectively. (**B**) Breast cancer cells were treated with either 5 (Hs578T) or 10 (BT549) μM of DAC every 24 hours for 5 days; while, controls cells were cultured in their respective basal media. (**C**) BT549 breast cancer cells were treated with either Entinostat (MS275, 2 μM) or DMSO (2 μl) for 24 hours; while, controls for each cell line were cultured in the respective basal media. Genomic DNA from Hs578T and BT549 cells was treated, with Sodium bisulfite, and used as a template for PCR. Amplicons generated were sequenced and the methylation status of the 17 CpG sites in the FRK promoter determined. Circles filled with red represent methylated CpG sites, while green and orange circles represent non-methylated and differentially methylated CpG sites, respectively. Treatment of Hs578T cells with DAC partially demethylated CpG 7 at site +6 from the TSS; while, treatment of BT549 cells with Entinostat demethylated CpGs 11 and 12 at site −258/−350 from the TSS.

In order to evaluate whether the reactivation of *FRK* expression in breast cancer cells by epigenetic drugs was associated with promoter CpG demethylation, BT549 and Hs578T were evaluated after 5 days of 24 hourly treatments with DAC, and in the BT549, 24 hours post-treatment with Entinostat (Figure 5A). Treatment with DAC partially demethylated CpG 7, at position +6 in Hs578T cells but had no effect in the BT459 cells (Figure 5B). Treatment of BT549 cells with Entinostat demethylated CpGs 11 and 12, at positions -258 and -350, respectively (Figure 5C). Based on our observation therefore promoter site-specific de-methylation of CpGs 11 and 12 is important for the up-regulation of *FRK* in breast cancer.

### The promoter regions proximal to CpGs 11 and 12 are crucial for the transcription of *FRK*

**Figure 6:**
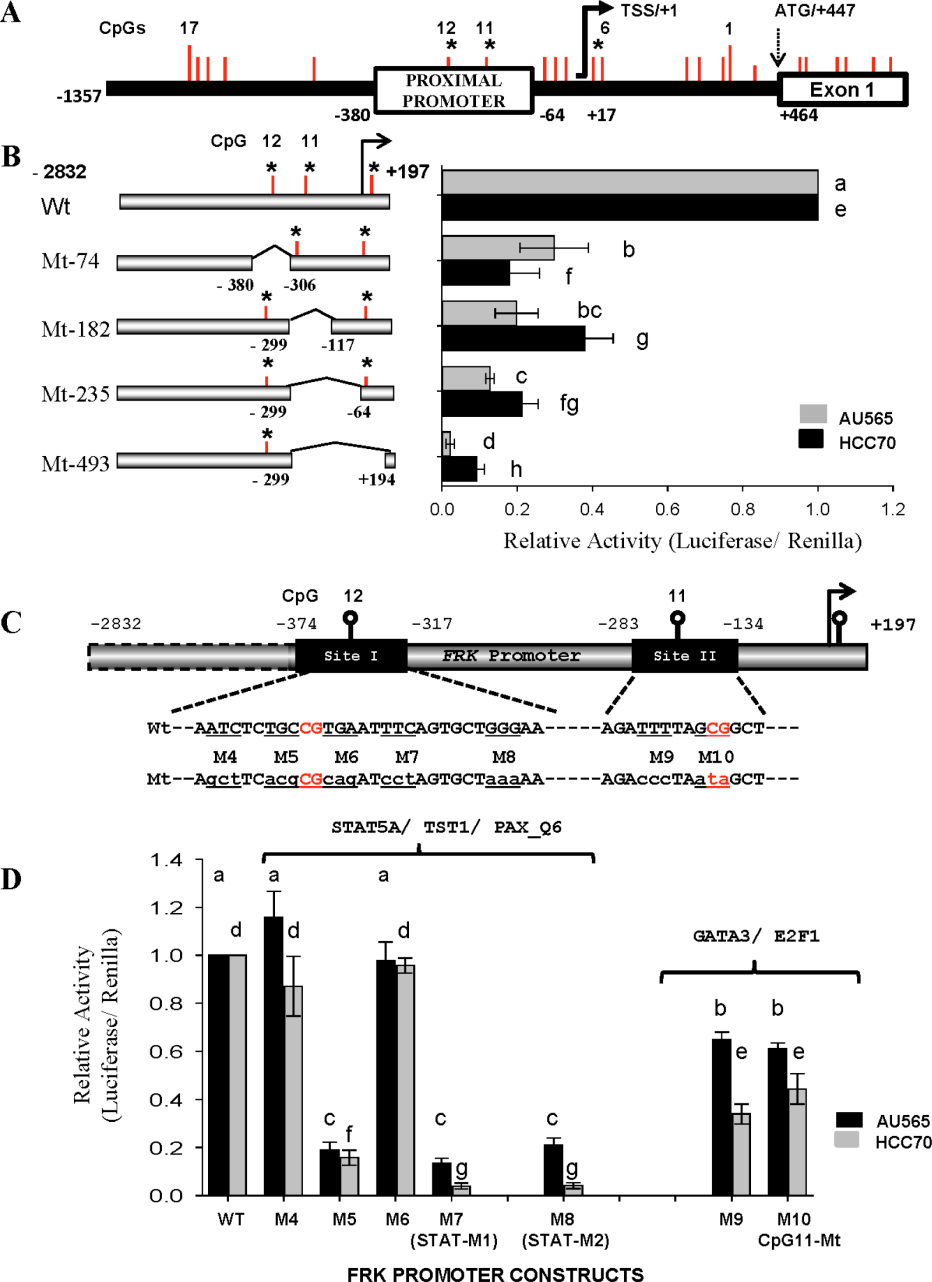
The promoter regions proximal to CpGs 11 and 12 are crucial for the transcription of *FRK* (**A**) A schematic representation of the *FRK* promoter region from +447 to −1357 of the TSS/+1 showing the 17 CpG sites as vertical red lines along the promoter. (**B**) The CpG sites 11 and 12 are marked as vertical red lines within each of the constructs. The region deleted from each promoter construct is indicated along the breaks of each horizontal bar to the left of the y-axis for constructs mutant 74 (Mt-74), Mt-185, Mt 235 and Mt-493. (**C**) A schematic representation of the *FRK* promoter regions flanking the CpG 12 and 11, sites I (−374/−317) and II (−283/ −134). The wild type (upper case) and the mutated nucleotides (lower case) are shown below each of the targeted sites. The human *FRK* promoter mutant constructs (Mt; M4 to M8 and M9 to M10 carried site-specific mutations as indicated in sequences below the site boxes (C). AU565 and HCC70 breast cancer cells were transfected with 495 ng per well of various *FRK* promoter constructs of the human FRK promoter as indicated on the left of the y-axis (B) or below the x-axis (**D**). Results are shown as relative activity of each construct with respect to the full length FRK promoter construct −2832/+197bp (mean ± S.E.M.). A different letter indicates a statistically significant difference.

Prior to this study, little was known regarding the transcriptional regulation of human *FRK*. Using the USCS database (http://genome.ucsc.edu/), we determined the *FRK* promoter region and TSS/+1. In order to know if the CpGs sites implicated above play a role in *FRK* transcription we cloned the *FRK* promoter fragment (−2832/+194bp) along with deletion constructs into a pGL3-basic vector (Promega Corporation; Figure 6). The reporter constructs were transiently transfected into AU565 and HCC70 cell lines, with high endogenous FRK levels. In both cells, promoter activity decreased following internal deletions of the *FRK* promoter (*P* < 0.05; Figure 6B). The decrease in activity indicated the presence of regulatory sites within the deleted regions, -380/306 and −299/−117 bp, that are crucial for the transcription of *FRK*.

The promoter regions −374/−317 and −299/+89 bp, defined as site I and II, respectively, were then interrogated for regulatory elements using the program Multi-genome Analysis of Positions and Patterns of Elements of Regulation (http://ecrbrowser.dcode.org; Figure 6C). The putative DNA binding sites for transcription factors GFI1, STAT5A, TST1 and PAX overlapped CpG 12 (Table 1). Site-mutations M5, M7 and M8 within region −374/−317 of the full-length FRK promoter decreased its activity in the AU565 and HCC70 cells (*P* < 0.05; Figure 6D). Mutations M5, M7 and M8 altered the canonical STAT binding sequence TTC-[N]_3–6_-GAA [42, 43].

**Table 1:** Putative binding sites proximal to CpG sites 11 and 12 identified by using *in silico* analysis

	Transcription factor	Putative sequence	Name of mutated sites
SITE I	GFI1	gggAAAAAA**ATC**TC**TG**CCGTGaat	M4, M5 and M6
	STAT5A	**tg**C**CGTGA**AT**TTC**AGTGCT**GGG**AA	M5, M6, M7 and M8
	TST1_01	cgtgAATttcagtgc	M6 and M7
	PAX_Q6	GTGAATTTCAG	M6 and M7
SITE II	E2F_02	TTTAGCGG	M10
	HNF6_Q6	atATAGATTTta	M9
	GATA3	ataGATTTTA	M9

The underlined sites indicate the nucleotides that were mutated in constructs transiently transfected in the breast cancer cells.

Analysis of site II revealed potential binding sites for E2F1, HNF6, and GATA3 proximal to CpG 11 (Table 1). Tri-nucleotide mutations M9 and M10, within the region −283/−134 of the full length FRK promoter decreased its activity in theAU565 and HCC70 cells (*P* < 0.05; Figure 6C and 6D). Mutation M9 altered the canonical GATA3 binding sequences WGATAR, where W = A or T and R = A or G [44]; while, mutation M10 altered the canonical E2F1 binding sequences 5′-TTTSSCGS-3′, where S = C/G [45]. Our data indicate that the DNA binding sites of STAT5A, E2F1, and GATA3 are crucial for FRK transcription.

### Constitutively active STAT5A up-regulates the *FRK* promoter activity

**Figure 7:**
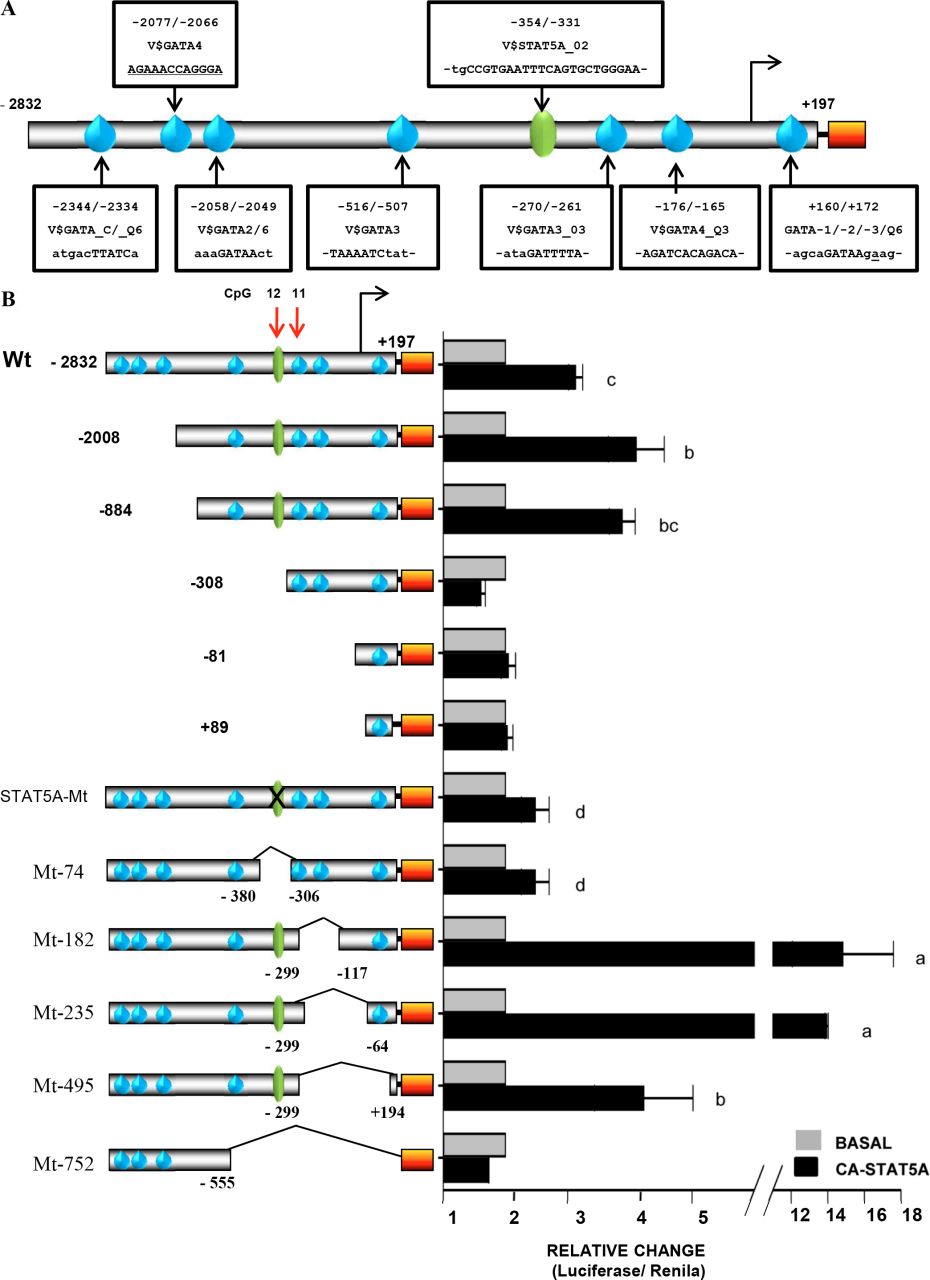
Constitutively active STAT5A up-regulates the *FRK* promoter activity (**A**) A schematic representation of the FRK promoter region −2832/+197, that was cloned up stream of the luciferase reporter gene (Orange box). The sequences of the putative binding sites of STAT5A and the GATA proteins (1, 2, 3, 4 and 6) are indicated along the promoter in boxes. (**B**) AU565 breast cancer cells were co-transfected with various *FRK* promoter constructs (245 ng per well) as indicated on the left of the y-axis in the graph 8B) along with 250 ng per well of a construct that express the constitutively active STAT5A(CA-STAT5A). Total DNA per well was kept at 500 ng per well. AU565 controls cells in each case were transfected with the empty pCDNA3 vector (Invitrogen). Results are shown as fold activation over control (mean ± SEM). The superscripts a-d indicates relative mean values that are significantly different (*P* ≤ 0.05).

To determine if the STAT5A-DNA binding site was biologically relevant, we examined the effects of STAT5A on *FRK* promoter activity by co-transfections in AU565 cells (Figure 7A). The activity of the full length *FRK* promoter construct increased by 3-4-fold in the presence of constitutively active STAT5A (CA-STAT5A, *P* < 0.05; Figure 7B). Co-transfection of the FRK promoter with either wild type (wt) STAT5A, Wt-STAT5B or CA-STAT5B had no effect on promoter activity (Supplementary Figure 6A). The FRK reporter constructs in which the STAT5A response element was either deleted (−308, −81, +89 and Mt-74) or mutated (STAT5A-Mt), were non-responsive to the CA-STAT5A (*P* > 0.05; Figure 7B). The transactivation potential of STAT5A was boosted by internal deletions in constructs Mt-182 and Mt-235 (Figure 7B). This increased response to CA-STAT5A indicates this region may contain negative regulatory elements of STAT5A trans-activation. STAT5A transactivates the *FRK* promoter through its putative DNA binding motif, −355/−331.We concluded that in breast cancer cells STAT5A up-regulates the transcriptional expression of *FRK*.

### Friend of GATA 1 up-regulates the *FRK* promoter activity

**Figure 8:**
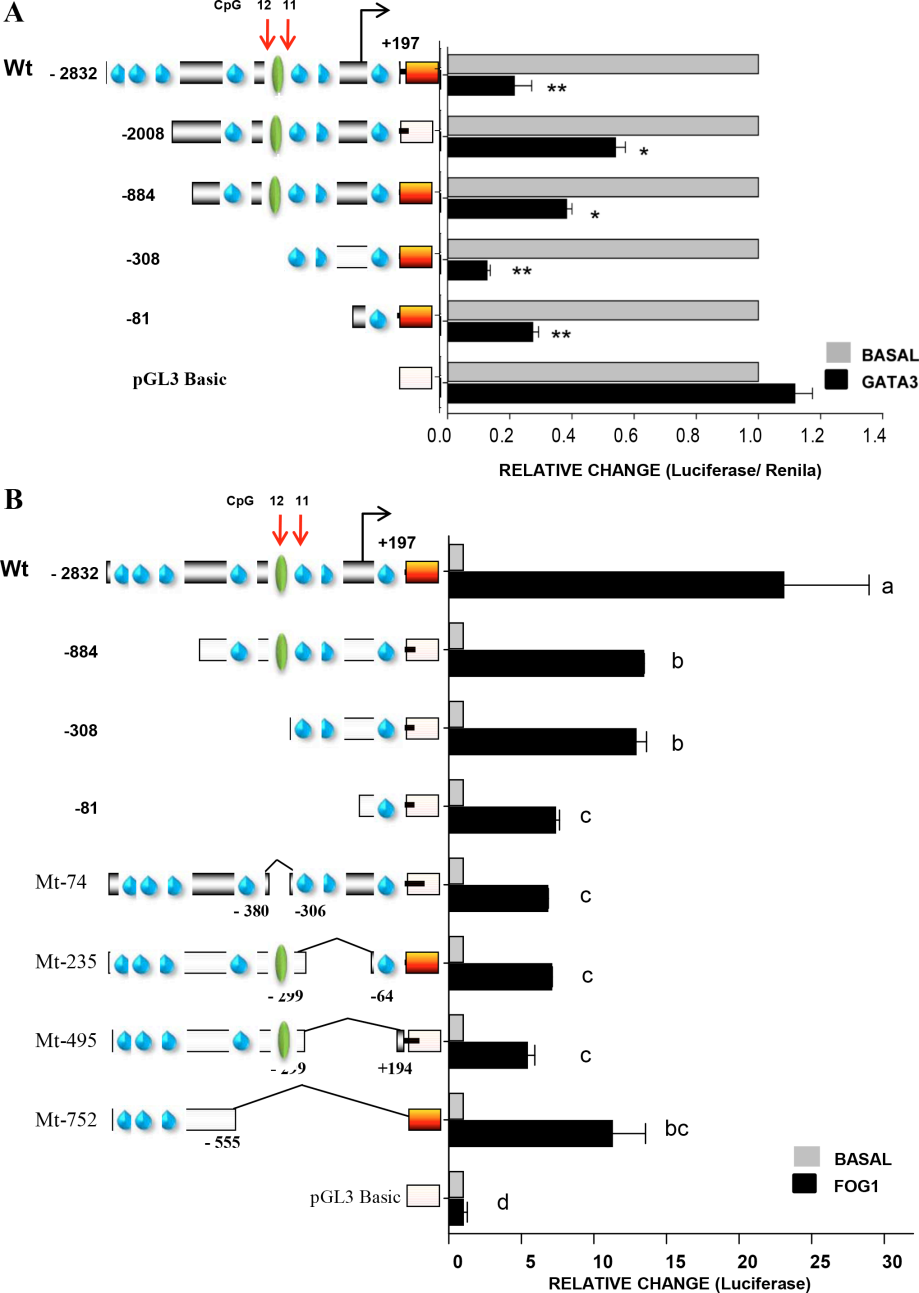
Friend of GATA 1 up-regulates the *FRK* promoter activity AU565 breast cancer cells were co-transfected with 245 ng per well of various *FRK* promoter constructs as indicated on the left of the y-axis in the graphs along with 250 ng per well of either the (**A**) GATA3 or (**B**) FOG1 expression vectors that code for one of the for the full length wild type proteins GATA3 and FOG1, respectively. AU565 controls cells in each case were transfected with the empty pCDNA3 vector (Invitrogen) and total DNA per well was kept at 500 ng. Results are shown as fold activation over control (mean ± SEM). The superscripts (a–d) indicate relative mean values that are significantly different (*P* ≤ 0.05).

Next, we investigated the biological relevance of the GATA-DNA binding site, also shown to be crucial for *FRK* transcription (Figures 6 and 7A). To this end, we co-transfected various FRK promoter constructs and the GATA3 construct into AU565 cells (Figure 7A). We found that the *FRK* promoter activity of all constructs was repressed by GATA3 (*P* < 0.05; Figure 8A), indicating the presence of other potential GATA response elements in the *FRK* promoter. GATA proteins were shown to be modulated by FOG-1 and 2 [46, 47]. Co-transfection of the *FRK* promoter with a vector coding the FOG-1 protein, resulted in the up-regulation all the FRK constructs except the empty pGL3-vector (*P* < 0.05; Figure 8B). Interestingly, deletion of the STAT5A binding region that span the CpG 12 site (Mt-74) significantly decreased the transactivation of *FRK* by FOG1 as was observed when the GATA3 binding region that spun CpG 11 site (Mt-235) was deleted (Figure 8B). These findings indicate that the co-modulator of GATA3, FOG1, up-regulates *FRK* by overcoming the GATA3 driven repression of FRK.

## DISCUSSION

The role of FRK in human cancer is unclear; however some reports have shown that its expression is lost in breast cancers and that its re-expression suppresses breast tumor growth [17]. To understand the expression pattern of FRK in breast cancer, we examined the levels of the transcript and protein in a panel of 40 human breast cancer cell lines and 4 non-malignant mammary epithelial cell lines. FRK levels were low or absent in about 20% of the cell lines, 6 of the 9 with low FRK levels belonged to the basal B category, 2 were classified as luminal and 1 basal A.

Epigenetic changes are one of the most important molecular aberrations in the pathogenesis of cancer [24, 48]. Epigenetic repression is often associated with genes where the promoter has a high density of CpG sites (CpG-islands); however, the *FRK* promoter has a low CpG content with 27 CpG sites spanning 3223 bp, from -2832 to +391. We determined the methylation status of the 17 CpG dinucleotides in the *FRK* promoter (+391/−959 bp) for 19 cell lines. With the exception of the HCC1419 cell line, the *FRK* promoter was highly methylated in breast cancer cell lines with low *FRK* expression. Methylation was largely absent in cell lines with high FRK, with exception of the breast cancer cell lines ZR-75-1 (Figure 2B) and CAMA-1 (Supplementary Figure 2). In breast cancer cells, ZR-75-1 and CAMA-1 the promoter region (+391/−959) was densely methylated even though the FRK (mRNA and protein) expression was high. However, the absence of methylation at CpGs 12 and 11 at sites -350 and -258 respectively was consistent in the two cell lines (ZR-75-1 and CAMA-1) with other high *FRK* expressing cell lines. We further performed in-*silico* analysis of breast cancer patient data from the Cancer Genome Atlas (TCGA) and established that the *FRK* proximal promoter region in tumors samples had a lower methylation density compared with the matched normal tissues (Supplementary Table 8 and Supplementary Figure 8A, 8B) [49]. Observations from both breast cancer cells and TCGA data led to the conclusion that the transcription repression of *FRK* was independent of the FRK promoter methylation density. Interestingly, two sites, CpG 11 and 12 at sites -258 and -350 from the TSS/+1, were methylated in the 9 of 36 cells with an incidence of 27.8 %. However, CpG 11 site was more prone to hemi-methylation (16.7 %). All the 9 cell lines exhibited low FRK expression levels. It was apparent that methylation of sites 11 and 12 was associated with decreased FRK expression, suggestive of site-specific methylation. Site-specific methylation was previously cited as the mechanism behind *hTERT* and *ANKRD11* repression in colorectal carcinoma and breast cancer, respectively [50].

Treatment of cancer cells with epigenetic drugs alters the chromatin landscape resulting in the differential expression of genes [51–54]. DAC, a demethylating agent, was reported to reactivate silenced genes by passively inhibiting the activity of DNMT [38]. The HDI, TSA acted in synergy with DAC in the induction of retinoic acid receptor (*RAR*) βeta and estrogen receptor-1 expression in MDA-MB-231 cells [55]. Inhibition of HDAC activity by HDIs such as TSA, Entinostat and Mocetinostat results in the re-expression of tumor suppressor genes [52–54]. In our preliminary studies, treatment of MDA-MB-231 cells with DAC in combination with TSA, led to a lower induction of *FRK* as compared to DAC only (Supplementary Figure 4). DAC induced the expression of *FRK* in all the cell lines; however, in the BT549 cells unlike the other cell lines there was no increase in FRK protein levels. The decreased FRK protein levels in the BT549 cells could have been due to a drug induced translational defect. It was suggested that high concentrations of DAC were incorporated in both DNA and RNA leading to the formation of defective tRNAs and rRNAs thereby inhibiting protein synthesis [56].

Treatment with DAC resulted in a partial demethylation of CpG 7 at position +6 in Hs578T cells but had no effect in BT549. It is therefore likely that DAC could have induced the expression of *FRK* without demethylating any of the CpG sites in the promoter region as was noted when the hypomethylated BT20 breast cancer cells were treated (Supplementary Figures 2 and 4). Decetabine was shown to up-regulate, *CDKN1A* and *CDKN2D* independent of promoter CpG demethylation [57, 58]. The increased expression of *CDKN1A* was linked to the demethylation of an upstream regulator P73, while activation of *CDKN2D* was thought to be a consequence of global gene demethylation [57, 58]. Interestingly, treatment of the hypomethylated SKBR3 cells with DAC led to the down-regulation of *FRK* (Supplementary Figure 4). The response to DAC was cell specific.

Treatment of breast cancer cells with Entinostat and Mocetinostat resulted in 10 to 33-fold increase of *FRK* levels. The increase in FRK mRNA was translated into higher protein levels in BT549 cells. On evaluating the *FRK* promoter methylation status in the BT549 cells 24 hours post-treatment with Entinostat, CpGs 11 and 12, at positions -258 and -350, were demethylated. HDIs, TSA, depsipeptide and sodium butyrate, were shown to enhance the expression of genes such as *CDKN2A*, *CDKN1A*, *SALL3*, *RARb2, TERT* and *GATA4* in human cancer cell lines by active DNA demethylation of their respective promoters [39–41]. In addition, depsipeptide decreased the binding of DNMT1 to the promoter, while sodium butyrate repressed MAP kinase I, down regulating the cellular levels of DNMT1 [39, 40]. Interestingly, treatment of the hypomethylated HCC1419 cells with Entinostat and Mocetinostat also increased *FRK* expression (Supplementary Figure 4). It is likely Entinostat induced the expression of FRK via mechanisms other than promoter DNA demethylation. Treatment of BT549 with Entinostat displaced the chromatin repressive markers histone H3K9me3 and H3K27me3 from the *FRK* promoter (Supplementary Figure 5). Entinostat was shown to increase H4 acetylation [59]. Acetylation of histone H4 plays a primary role in the structural changes of the chromatin enhancing binding of transcription factors to their recognition sites within nucleosomes [60].

Prior to this study there was no report detailing the transcriptional regulation of *FRK* in humans, therefore the significance of these methylated sites was unclear. Promoter regions that were crucial of the *FRK* transcription activity were mapped using internal deletions, −380/−306 and 299/−117 that contained the CpGs 12 and 11, at site −350 and −258, respectively. We determined that the DNA sequences proximal to CpG 12 and 11 contained binding sites for specific transcription factors. Of great interest were sequences tgCCGTGAATTTCAGTGCTGGGAA and ataGATTTTA that span the promoter regions −354/−331 and −270/−261. These *FRK* promoter sequences were consistent with the canonical binding sites for STAT5 (−354/−331) and GATA (−270/−261), TTC-[N]_n_-GAA and WGATAR, respectively [42–44].

In this study, constitutively active (CA) STAT5A transactivated the *FRK* promoter constructs with the putative STAT5A response element; however, STAT5B had no effect (Supplementary Figure 6A). It was suggested that a spacer (n) of greater than 4 nucleotides favored binding of STAT5A over STAT5B [42, 43]. The *FRK* sequence from -354 to -331 of the TSS/+1 has a spacer of 7 nucleotides. Mutations (M7 and M8) and deletions of the sequence −354/−331 ablated the transactivation of the promoter by CA-STAT5A in AU565 cells. Interestingly, Entinostat and Mocetinostat up-regulated the expression of *STAT5A/B* along with *FRK* levels (Supplementary Figure 5). It is likely that methylation CpG 12 interferes with binding of STAT5A to its DNA sequence on the *FRK* promoter hence repressing *FRK* expression in the breast cancer cells. Methylation of the STAT5A DNA-binding sequence in the αS1-casein promoter was reported to repress the prolactin induced expression of the αS1-casein during acute mastitis [61]. We noted that the transactivation potential of CA-STAT5A was boosted by deletions that span the CpG11 (constructs Mt-182 and Mt-235). It's therefore likely that the deleted sequence harbored a STAT5A repressor element. We established the region spanning the CpG 11 at site -258 contained putative binding sequences for the GATA- and E2F- family of transcription factors, denoted as WGATAR, where W = A or T and R = A or G [44] and 5′-TTTSSCGS-3′, where S = C/G, respectively [45]. Mutations M9 and M10 within the core sequence ATTTTAGCGG in the full-length promoter ablated the *FRK* promoter activity. The effectors coding for the full-length proteins GATA-3 and E2F-1 repressed the activity of the *FRK* promoter (Figure 8A and Supplementary Figure 6B). This could explain why deletion of this region −299/−64 led to the increased response of the FRK promoter to CA-STAT5A.

The activity of GATA proteins was previously shown to be modulated by FOG 1 and 2 [46, 47, 62]. Co-transfection of the *FRK* promoter with a vector encoding the full length FOG1 protein, up-regulated the *FRK* promoter activity of constructs with functional GATA sites. Interestingly, FOG-2 had no effect on the *FRK* promoter activity (data not shown). It is worth noting *in-silico* analysis of protein-protein interaction networks using STRING v9.1 (http://string-db.org/) shows a potential interaction of STAT5A with FOG1 but not FOG2 [63]. This could explain why deletion of the STAT5A binding region that spans CpG 12 site (Mt-74) led to the decreased transactivation of *FRK* by FOG1 as was observed when the GATA3 binding region that span CpG 11 was deleted. We concluded that the repression of FRK in a subset of breast cancer cell lines was a consequence of methylation at specific CpG sites, -258 and -350. It is likely that methylation of CpG 11 and 12 interfered with binding of STAT5A and GATA3 to their putative core sequences, with the repressive actions of the GATA3 reversed by FOG1. In this study, the basal expression of *FRK* across all the 40 cells was not correlated with the expression of either *STAT5 (A/B)* or *GATA3/FOG1* (Supplementary Figure 7A–7D). It is worth noting that, with the exception of the Hs578T cells, all the low *FRK* expressing cell lines had very low levels of *STAT5A*, although this phenomenon was not exclusive to *FRK*-low cells. High *FRK* expressing cell lines like UACC893, ZR-75-1, MDA-MB-361, MDA-MB-175 and HCC1569 had low STAT5A levels. While GATA-3 expressions were also low in all the *FRK*-low breast cancer cells, with exception of MDA-kb2 and the HCC1419, likewise this was not limited to the *FRK*-low cells.

Our findings support the association of site-specific methylation of the *FRK* promoter with its reduced expression in breast cancer cells. The loss of expression was more prevalent in the basal breast cancer cell type. This suggests that loss of FRK expression may promote cancer growth or development in a particular cellular context. Our findings highlight the clinical relevance of epigenetic drugs in the re-activation of silenced tumor repressor genes in breast cancer.

## MATERIALS AND METHODS

### Cell culture

Breast cancer cell lines and non-tumorigenic cells derived from normal human breast epithelia were stratified into 3 different breast tumor phenotypes: Basal A (BA); Basal B (BB); and Luminal (LU) (Supplementary Table 1). All cells were cultured as recommended by American Type Culture Collection (ATCCA; Manassas, VA USA).

### Decitabine treatment

BT549, HCC1395, Hs578T and MDA-kb2 cell lines were seeded in 10 cm plates were treated with either 5 μM (BT549, Hs578T and MDA-MB-231) or 10 μM (HCC1395 and MDA-kb2) of DAC (Sigma-Aldrich, Ontario Canada), every 24 hours, over a selected period (1, 2, 3, 4, and 5 days). The culture media in all plates was replenished every 24 hours, prior to treatments with DAC. Concentrations of DAC used were determined using a dose response curve in which cells were treated with DAC every 24 hours for 3 days (Supplementary Figure 3). Treatment with DACwas initiated when cells attained a 70% confluence, cells were culturedfor 6 days and control cells were grown in their basic media.

### Entinostat and mocetinostat treatments

Breast cancer cell lines BT549, HCC1395 and Hs578T were seeded in 21 different 10 cm plates as above. The cell lines were treated with either Entinostat (2 mM) or Mocetinostat(1 μM) for 6, 12 and 24 hours, while the control cells for each treatment time point received the vehicle DMSO (0.2 mL/mL). Treatments were initiated when the cells attained 80% confluence.

### RNA isolation, reverse transcription, PCR and real time PCR

Total RNA was isolated from cells using Trizol^(R)^ as recommended by the manufacturer (Invitrogen Canada, Burlington, Ontario, Canada). cDNA was synthesized using the Thermo-scientific maxima first strand cDNA synthesis Kit (ThermoFisher Scientific, Lafayette, CO 80026, United States). The cDNA synthesized was used as a template in quantitative RT-PCR. The expression of *FRK* and house-keeping genes *GAPDH* and *RPL13A* was determined using TaqMan^R^ probes Hs00176619_m1; Hs275991 and Hs04194366, respectively as recommended by manufacturer (Thermo Fisher Scientific). *FRK* expression was normalized to that of the house keeping gene detected within the same well by an Applied Biosystems^TM^, Step One Plus qRT-PCR machine (Life Technologies, Burlington). The relative expression of *FRK* to the house keeping gene (*GAPDH* or *RPL13A*) of each sample was then determined with respect to that of HCC1395.

### Immunoblotting

Breast cancer cells were harvested and the FRK and β-actin protein expression in cell lysates detected as was previously published [64]. FRK was probed for using a mouse polyclonal antibody (Santa Cruz).

### Sodium bisulphate DNA modification and bisulphite sequencing

Genomic DNA was isolated from cells using the DNeasy, Blood and Tissue Kit as recommended by the manufacturer (Qiagen, Hilden, Germany). The DNA was treated with Sodium bisulfite using the EpiTect^R^Bisulfite Kit according to the manufacturer´s instructions (Qiagen, Hilden). The treated DNA (100 ng) was then used as a template in PCR reactions with bisulfite-PCR primers.

The *FRK* promoter region −1357 to +464 from the transcription site was analyzed using a bioinformatics tool [65]. Bisulfite-PCR primers were designed spanning the regions, +464/−502 and −541/−1112 of the *FRK* promoter TSS/+1 (Supplementary Table 2). PCRs were performed using TaKaRaEpiTaq^™^ HS, as recommended by the manufacturer (TAKARA BIO INC, Tokyo Japan). The amplicons were resolved on agarose gels, purified using the QIAquick Gel Extraction Kit gel-extraction kit (Qiagen, Hilden) and sequenced (Supplementary Table 2).

### Plasmids

A -3029 bp fragment (−2832/+197) of the 5′-flanking end of the *FRK* gene was generated from human genomic DNA by PCR using the primers listed in Supplementary Table 3. The deletions were generated from the *FRK* plasmid using primers listed in Supplementary Tables 3 and 4. All the fragments were cloned into the pGL3-basic plasmid (Promega Corporation). Site-directed mutations were introduced in the *FRK* promoter construct −2832/+197 bp using the QuikChange II XL mutagenesis kit (Stratagene, La Jolla, CA) and oligonucleotides listed in Supplementary Table 5. Plasmids were verified by sequencing (National Research Council Canada, Saskatoon, SK).The wild type and constitutively active STAT5 expression vectors were provided by Dr. Luc J Martin, University of Moncton, Moncton NB [66].

### Transfections and luciferase assays

Transfections of AU565 and HCC70 cells were performed in 24-well plates using the ViaFect^(™)^ as recommended by the vendor (Promega Corporation). Briefly, on the day before transfection, 125,000 cells were seeded with 500 μL of media per well in 24-well plates. Cells were then transfected with 500 ng per well: 495 ng of FRK-reporter construct (Firefly Luciferase) along with 5 ng of phRL-TK (Renilla Luciferase) as an internal control for transfection efficiency. The cells were harvested 40 hours post-transfection and the Dual-Luciferase Assay System used to measure luciferase activities with the GloMax^®^ 96 Microplate *Luminometer* (Promega Corporation). Data reported represents an average of at least three experiments. To evaluate the role of the effectors on the *FRK* promoter, cells were co-transfected with 250 ng of either the effector plasmid or an empty pCDNA3 vector with 245 ng the *FRK* reporters and 5 ng phRL-TK internal control per well (Invitrogen Canada).

### Statistical analysis

The data was analyzed by a one-way analysis of variance (ANOVA; SigmaStat Version 2.0, Jadel Corporation, San Rafael, CA, USA). Multiple range comparisons of paired means were done using a Fishers LSD test or the Newman-Keuls test. Level of significance was set at *P* < 0.05. Data is reported as mean ± SEM.

## SUPPLEMENTARY MATERIALS FIGURES AND TABLES



## Abbreviations

Please see Supplementary Table 7 for list of acronyms.
