# Supplementary material for: Repression of Fyn-related kinase in breast cancer cells is associated with promoter site-specific CpG methylation
Source: Oncotarget. 2017 Jan 6;8(7):11442–59. doi: 10.18632/oncotarget.14546 (PMC5355277; doi:10.18632/oncotarget.14546)
Supplement: Supplementary file 1 [file oncotarget-08-11442-s001.pdf]

Repression of Fyn-related kinase in breast cancer cells is associated with promoter site-specific CpG methylation

Supplementary Materials

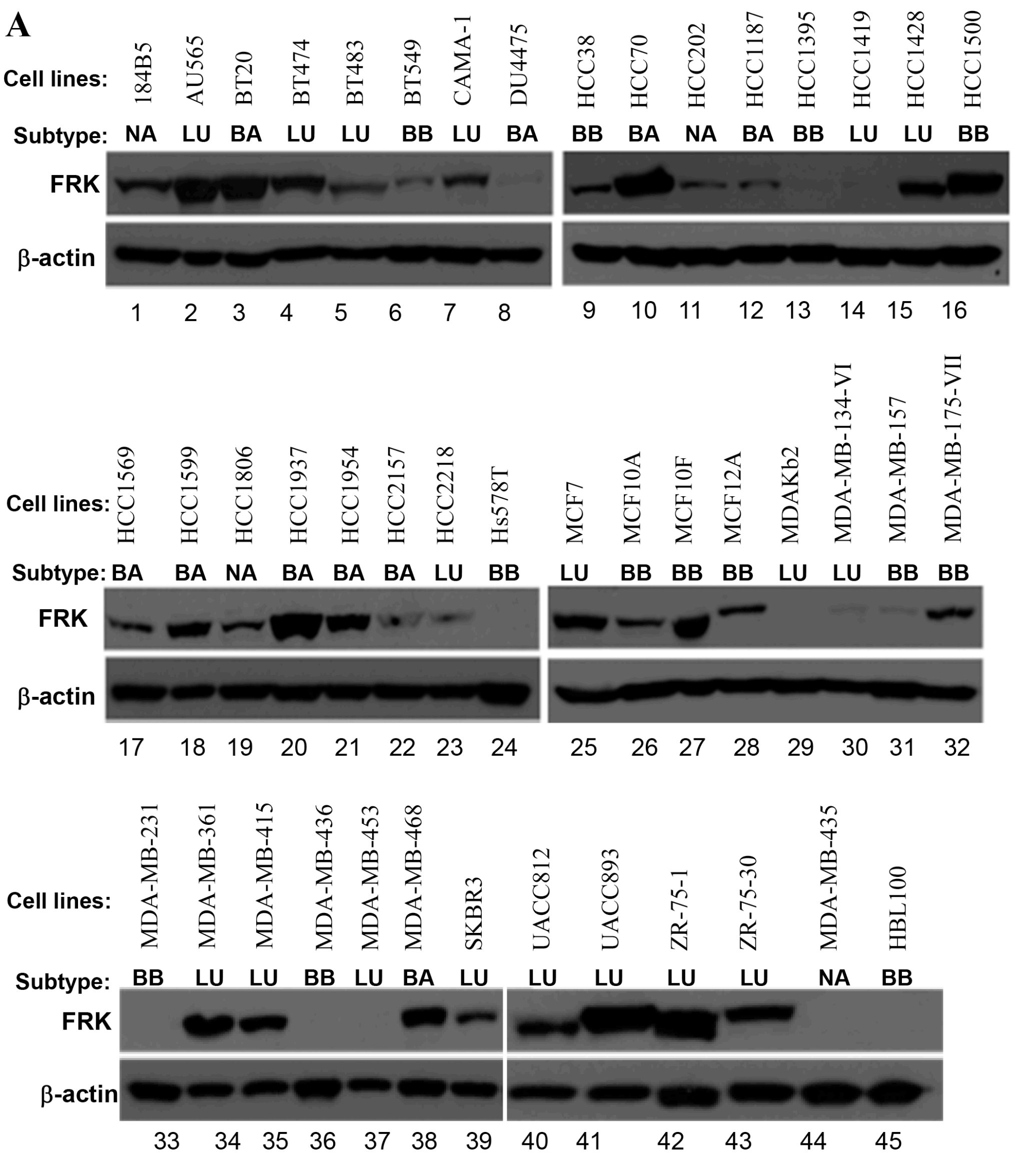

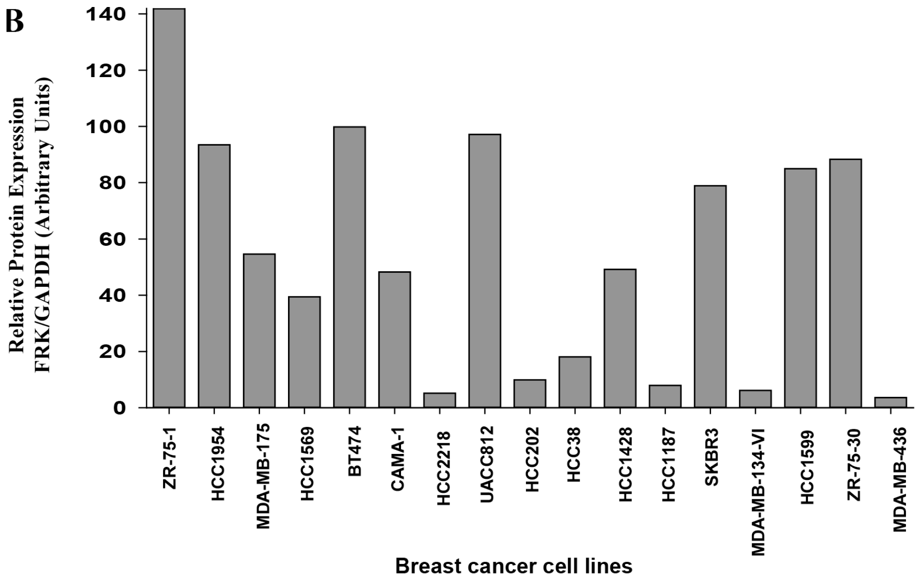

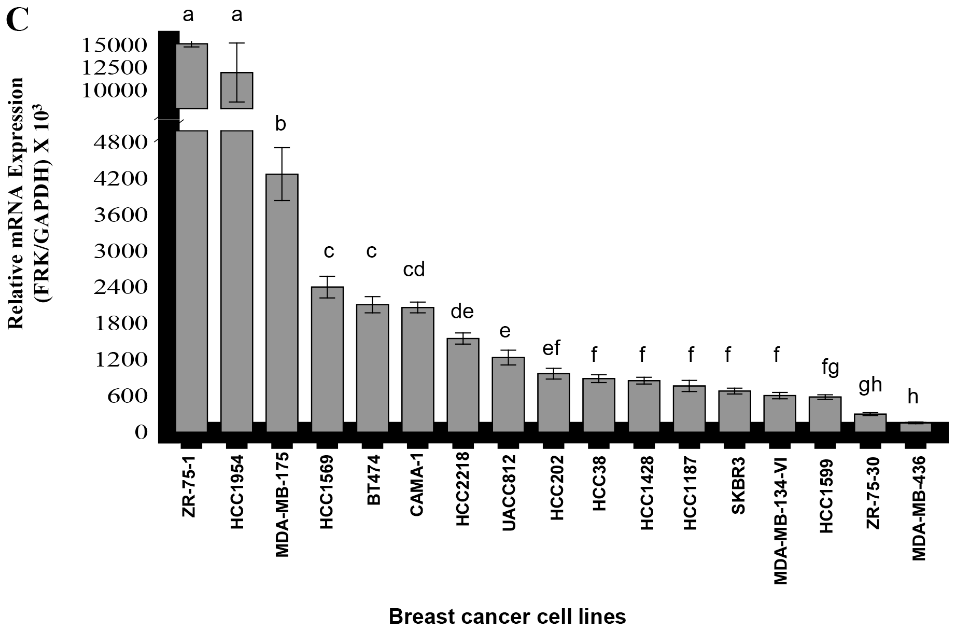

Supplementary Figure S1: Expression levels of FRK mRNA and protein in breast cancer cells. (**A**) The FRK protein levels in a panel of 45 human breast cancer cell lines was analyzed from the cell lysates by immuno-blotting using an FRK antibody (Santa Cruz CA), beta-actin, a house keeping gene was used as a loading control (A) and the mean relative protein expression levels were then determined by densitometry and expressed using arbitrary units (**B**). The FRK transcript levels were determined relative to that of GAPDH in each breast cancer cell were assessed by qRT-PCR and then normalized to that of the HCC1395 breast cancer cell line in order to determine the relative FRK transcript abundance. The data is presented as Mean ± SEM, different superscripts a-z are used to indicate significant differences across mean transcript levels (a-z = $P \leq 0.05$; Supplementary (**C**) NA, not applicable; LU, luminal; BB, basal B; BA, basal A.

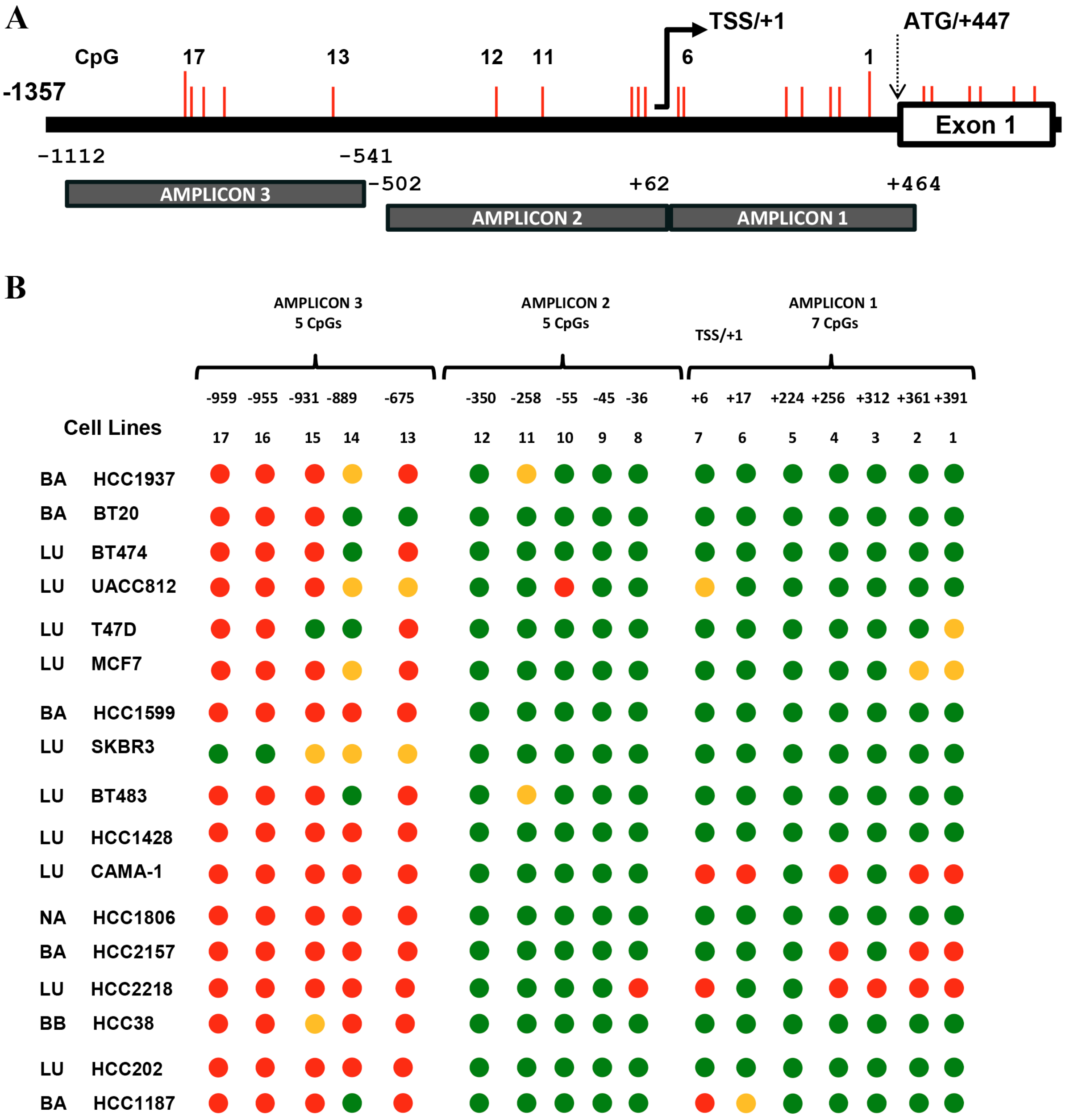

Supplementary Figure S2: The *FRK* promoter region is variably methylated in breast cancer cells. (**A**) A schematic representation of the FRK promoter region up-stream of exon 1, showing CpG sites as vertical red lines. Multiple methylation specific primers were designed spanning the 2 regions, +464/−502 and −541/−1112 of the 5′ un-translated region (UTR) and the non-coding region up-stream up stream of exon 1 and the FRK translation ATG-start site, using a bioinformatics tool[65]. (**B**) The methylation status of 17 CpG sites, numbered 1 to 17, from the translation start site (ATG) at site +391 to −959 bp of the transcriptional start site (TSS /+1) was determined. Genomic DNA was extracted from breast cancer cell lines with either low or high FRK mRNA expression and treated with sodium bisulfite, the DNA sequence of each amplicon was then determined to evaluate the methylation status of each of the 17 CpGs in the FRK promoter region numbered from +391 to −959 of the TSS (+1). Red, green and Orange circles represented the methylated, non-methylated and differentially methylated CpG sites, respectively.

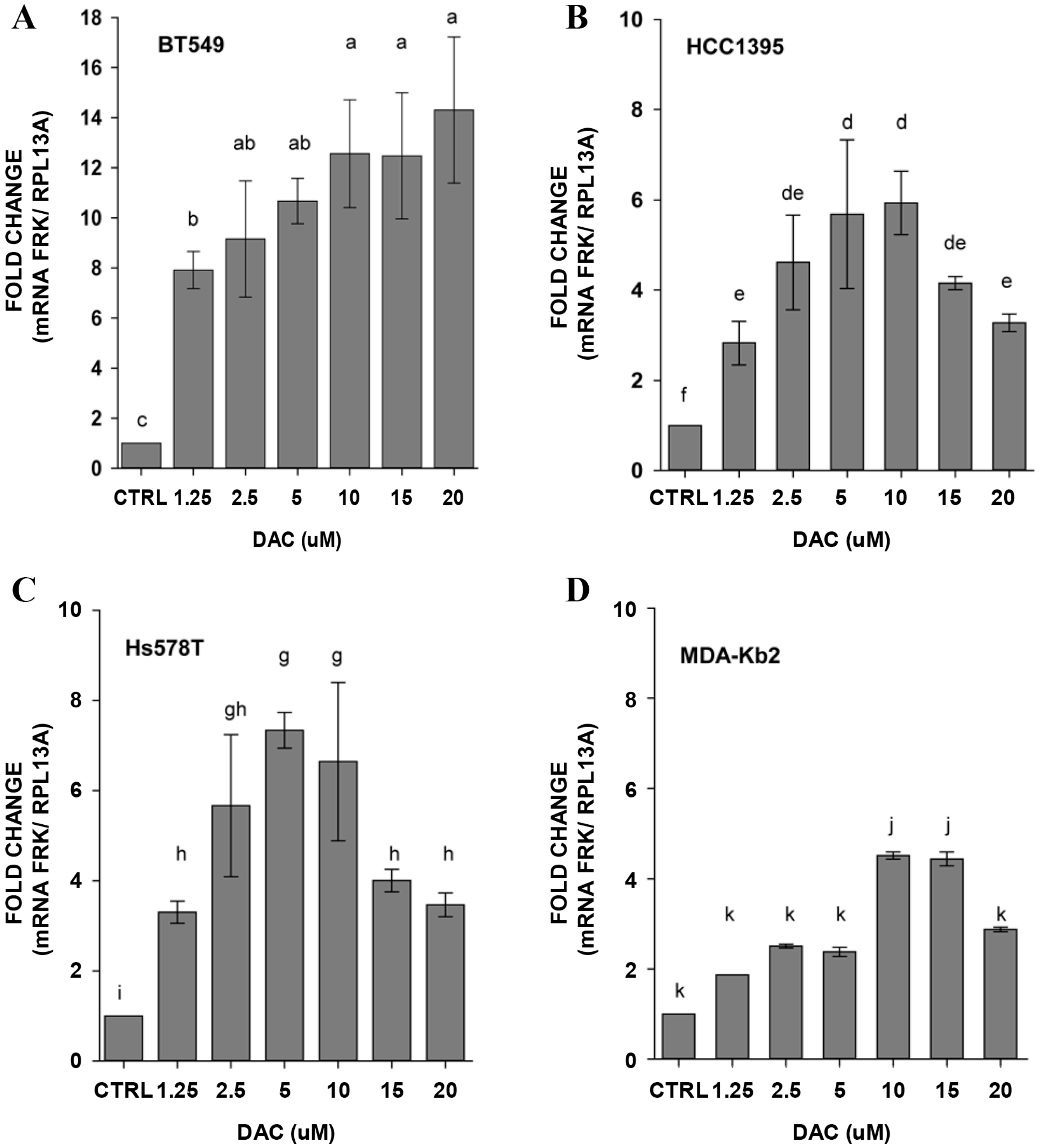

Supplementary Figure S3: Dose response curve in breast cancer cells following treatment with increasing concentrations of decitabine (DAC). Breast cancer cells, BT549 (**A**), HCC1395 (**B**), Hs578T (**C**) and MDA-Kb2 (**D**) were treated with increasing concentrations of DAC as indicated on the x-axis, every 24 hours, over a period of 3 days and the *FRK* transcript levels determined. FRK mRNA was extracted and quantified by qRT-PCR, FRK expression was determined relative to the GAPDH, then the fold change calculated relative to the controls. Data is presented as Mean ± SEM, * = mean values greater than controls $P \leq 0.05$.

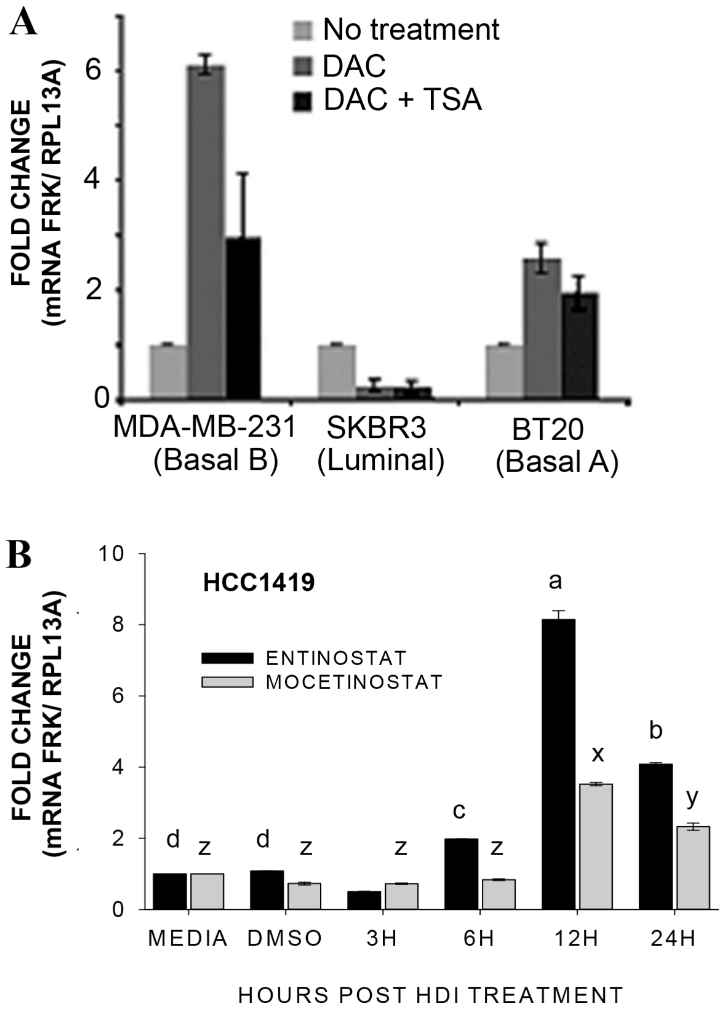

Supplementary Figure S4: Epigenetic drugs induce the expression of FRK in breast cancer cells. (**A**) Effect of DNA methyl transferase inhibition on the re-expression of *FRK*. Reactivation of FRK mRNA expression in breast cancer cell lines, MDA-MB-231 (Basal B), SKBR3 (Luminal) and BT20 (Basal A) with demethylating agent decitabine (DAC) and histone deacetylase inhibitor trichostatin A (*TSA*). The cells were treated with 4 uM DAC alone for 72 hours, followed by the presence or absence of 1 uM TSA for 24 hours. mRNA was extracted and quantified by qRT-PCR (* represent different mean values greater than controls $P \leq 0.05$). (**B**) The triple negative HCC1419 were treated with either Entinostat (MS275, 2 μM) or Mocetinostat (MGCD0103, 1 μM) over the time periods indicated on x-axis while controls received the vehicle. The FRK expression was determine relative to the RPL13A. The fold change was then calculated relative to the controls in each experiment. Data is presented as Mean ± SEM. Superscripts represent different mean values, a-e = $P \leq 0.05$.

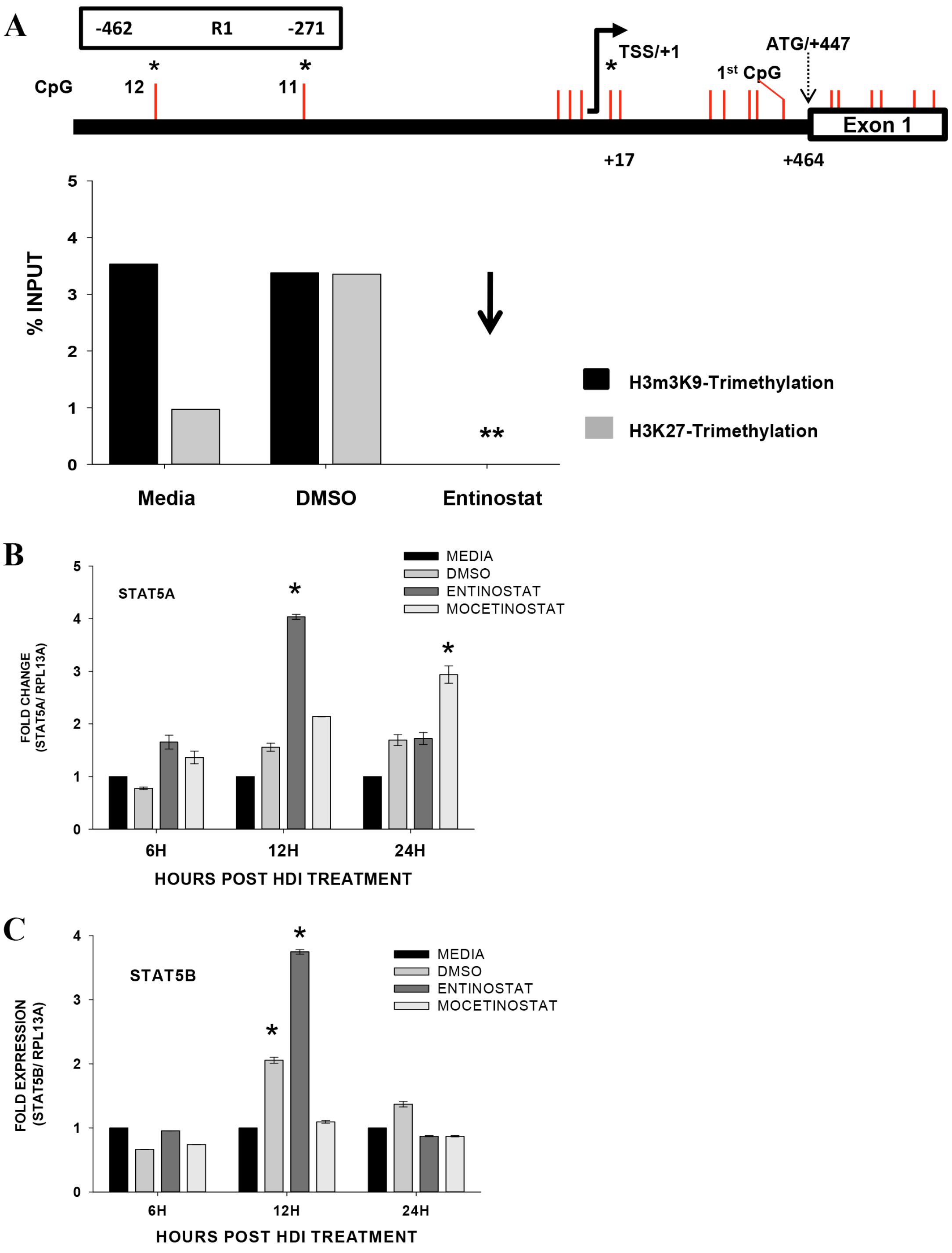

Supplementary Figure S5: FRK transcriptional repression is due to specific repressive chromatin marks. (**A**) Schematic representation of the FRK promoter region indicating the Region R1, −462 to −271, targeted for ChIP analysis with primers shown in Supplementary Table 6. Quantitative ChIP analysis was performed on BT549 breast cancer cells after treatment with Entinostat (MS275, 2 μM) for 24 hours, control cells were treated with vehicle (DMSO). The chromatin was incubated with antibodies directed againsttrimethylated H3K9 and H3K27 (Abcam). The immunoprecipitated DNA was then used as template in qPCR reactions using primers pairs targeting the *FRK* promoter region R1 (Supplementary Table 6). HDAC inhibitors Entinostat (MS275) and Mocetinostat (MGCD0103) transiently induces the expression of STAT5A (**B**) andSTAT5B (**C**) in breast cancer cells. The BT549 basal B breast cancer cells were treated with either Entinostat(MS275, 2 μM) or Mocetinostat (MGCD0103, 1 μM) over the time periods indicated on x-axis, while controls received the vehicle (DMSO). The mRNA was extracted and quantified by qRT-PCR, FRK expression was determine relative to the RPL13A, then the fold change calculated relative to the media controls. Data is presented as Mean ± SEM, * = mean values greater than controls $P \leq 0.05$.

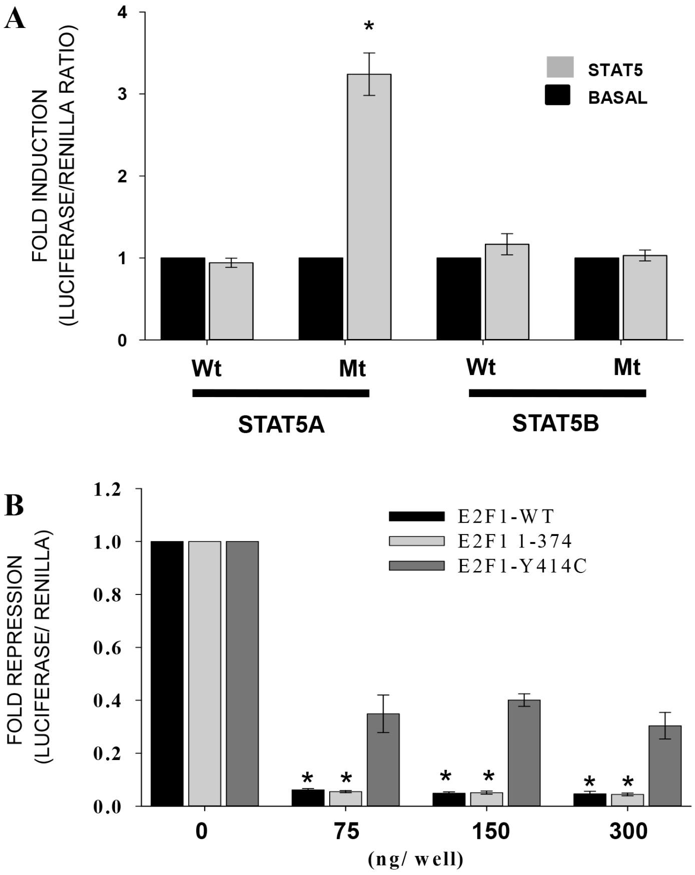

Supplementary Figure S6: *FRK* promoter activity is up-regulated by the constitutively active STAT5A and repressed by E2F1. AU565 breast cancer cells were co-transfected with the full-length promoter *FRK*, +2832/+197 along with effectors plasmids encoding either (**A**) STAT5 (STAT5A, CA-STAT5A, STAT5B and CA-STAT5B) or (**B**) the E2F-1 (E2F1-WT, E2F1-1-374 or E2F1-Y414C) and the promoter activity determine relative to the controls transfected with the empty pCDNA3 vector. Results are shown as fold activation over control (mean ± SEM). The Data is presented as Mean ± SEM, asterisks represent mean values greater than controls (*$P \leq 0.05$ and **$P \leq 0.01$).

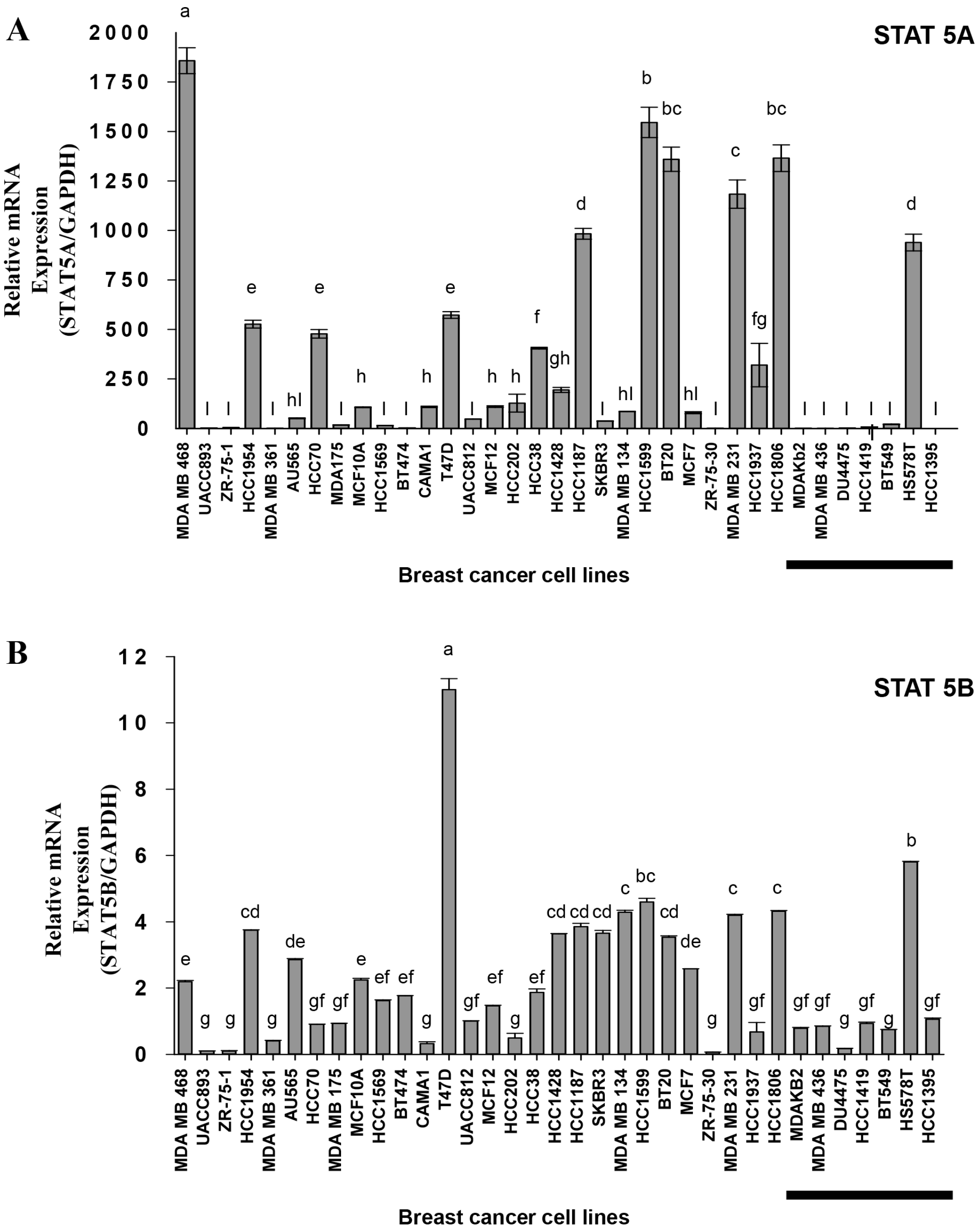
A
STAT 5A
Relative mRNA Expression (STAT5A/GAPDH)
0
250
500
750
1000
1250
1500
1750
2000
MDA MB 468
UACC893
ZR-75-1
HCC1954
MDA MB 361
AU565
HCC70
MDA175
MCF10A
HCC1569
BT474
CAMA1
T47D
UACC812
MCF12
HCC202
HCC38
HCC1428
HCC1187
SKBR3
MDA MB 134
HCC1599
BT20
MCF7
ZR-75-30
MDA MB 231
HCC1937
HCC1806
MDAKb2
MDA MB 436
DU4475
HCC1419
BT549
HS578T
HCC1395
Breast cancer cell lines
B
STAT 5B
Relative mRNA Expression (STAT5B/GAPDH)
0
2
4
6
8
10
12
MDA MB 468
UACC893
ZR-75-1
HCC1954
MDA MB 361
AU565
HCC70
MDA MB 175
MCF10A
HCC1569
BT474
CAMA1
T47D
UACC812
MCF12
HCC202
HCC38
HCC1428
HCC1187
SKBR3
MDA MB 134
HCC1599
BT20
MCF7
ZR-75-30
MDA MB 231
HCC1937
HCC1806
MDAKB2
MDA MB 436
DU4475
HCC1419
BT549
HS578T
HCC1395
Breast cancer cell lines

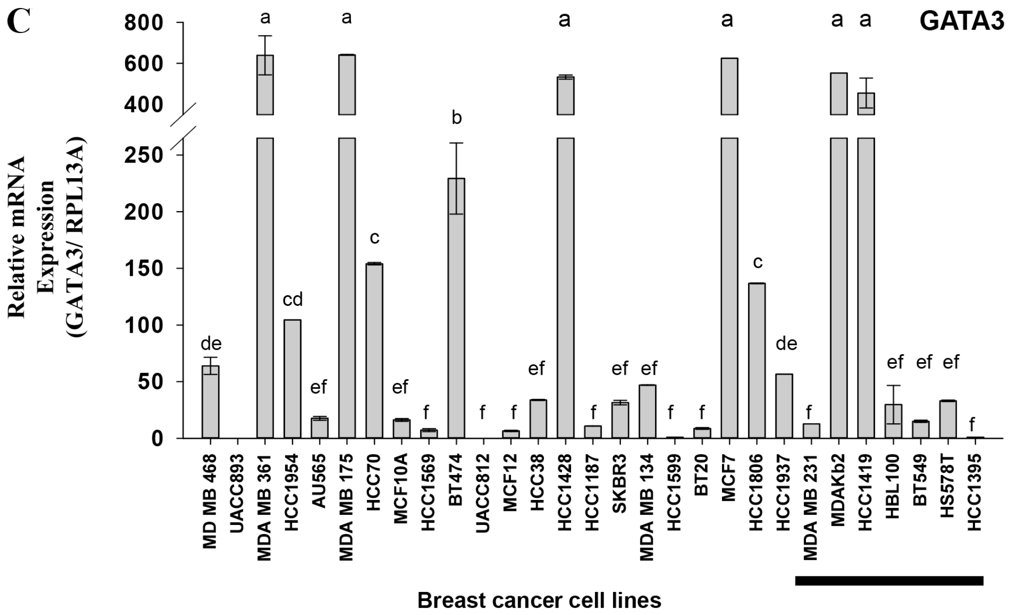

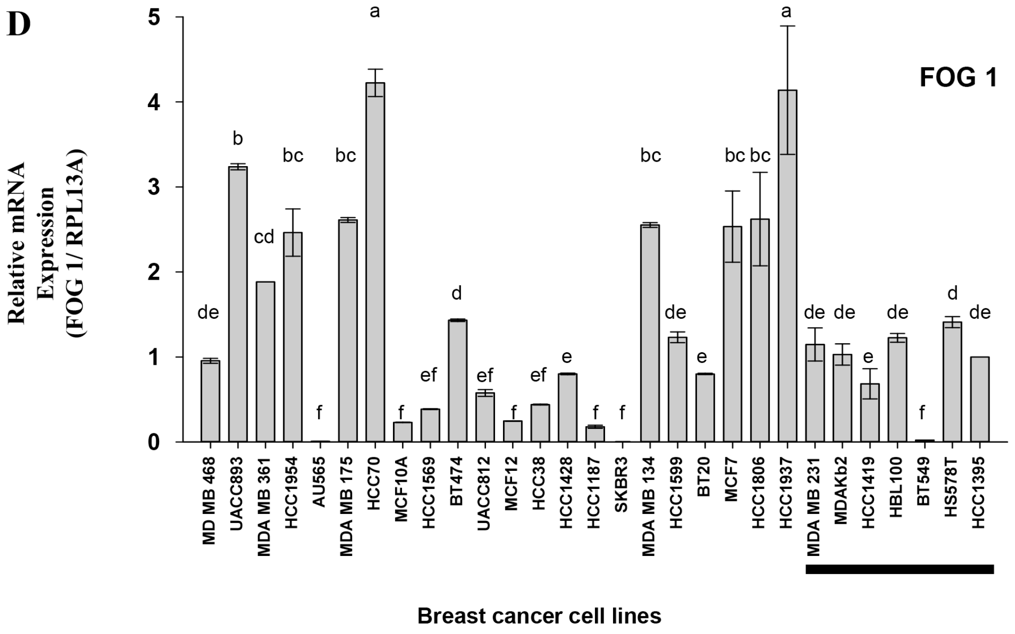

Supplementary Figure S7: Transcript levels of STAT5 (A/B), GATA3 and FOG1 in a subset of human breast cancer cells. The transcript levels of *STAT5A* (**A**), *STAT5B* (**B**) *GATA-3* (**C**), and FOG-1 (**D**) were determined relative to either *GAPDH* (A and B) or *RPL13A* (C and D) in each breast cancer cell using quantitative Real Time PCR. The relative expression of each cell was then normalized to that of the HCC1395 breast cancer cells in order to calculate the relative transcript abundance. Data is presented as mean ± SEM, superscripts are used to indicate the significant differences between means (a-z = $P \leq 0.05$). The cell lines with low FRK expression levels are shown using a horizontal line below the x-axis label stretching from HCC1395 to either MDA-kb2 (A and B) or MDA-MB-231 (C and C).

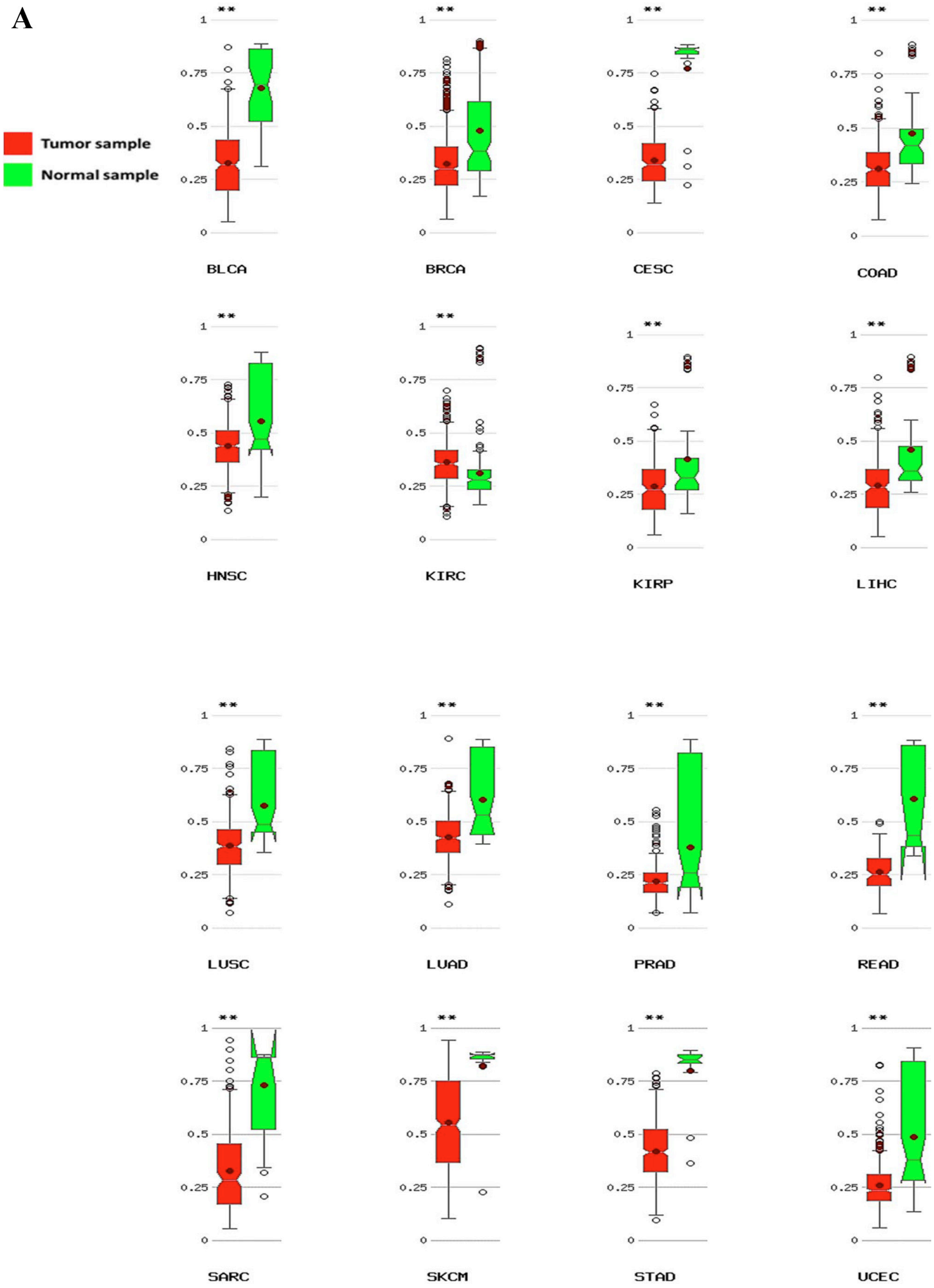
A
Tumor sample
Normal sample
BLCA
BRCA
CESC
COAD
HNSC
KIRC
KIRP
LIHC
LUSC
LUAD
PRAD
READ
SARC
SKCM
STAD
UCEC

B

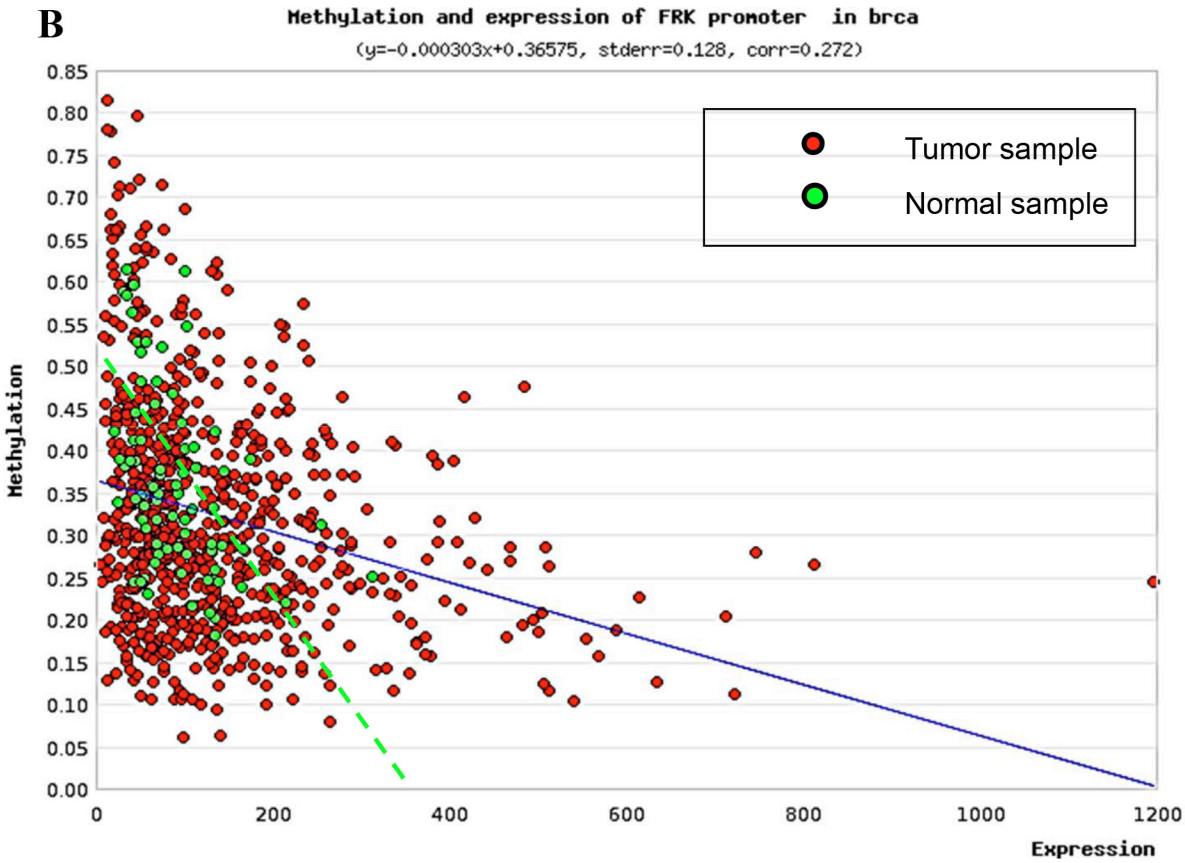

Supplementary Figure S8: The CpG methylation density of the FRK proximal proximal promoter region is lower in the different cancers types as compared to the normal tissue. The level of CpG methylation across the FRK proximal promoter (from −1500 bp to TSS+1) upstream of the first exon (8A) and the change in *FRK* expression with the degree of the its promoter CpG methylation (8B) was analyzed using an in-silico bio-informatics software MethHC[49]. MethHC integrates DNA methylation, gene expression, and the regression analysis of methylation with gene expression using data from TCGA (The Cancer Genome Atlas). Methylation across the FRK proximal promoter was determined in different tumors type (Red) and the paired normal tissue (Green). Promoter methylation was analyzed in different tumour types; Bladder Urothelial Carcinoma (BLCA), Breast Invasive Carcinoma (BRCA), Cervical Squamous Cell Carcinoma and Endocervical Adenocarcinoma (CESC), Colon Adenocarcinoma (COAD), Head and Neck Squamous Cell Carcinoma (HNSC), Kidney Renal Clear Cell Carcinoma (KIRC), Kidney Renal Papillary Cell Carcinoma (KIRP), Liver Hepatocellular Carcinoma (LIHC), Lung Adenocarcinoma (LUAD), Lung Squamous Cell Carcinoma (LUSC), Pancreatic Adenocarcinoma (PAAD), Prostate Adenocarcinoma (PRAD), Rectum Adenocarcinoma (READ), Sarcoma (SARC), Skin Cutaneous Melanoma (SKCM), stad: Stomach Adenocarcinoma (STAD), Thyroid Carcinoma (THCA), and Uterine Corpus Endometrial Carcinoma (UCEC). In Supplementary Figure 8A, the data is presented as Mean ± SD, asterisks represent mean values greater than controls (*$P \leq 0.05$ and **$P \leq 0.01$). In Supplementary Figure 8B, the variation of *FRK* gene expression with its promoter methylation analyzed in Breast invasive Carcinomas only (BRCA).

Supplementary Table S1: Human breast cancer cell lines and non-tumorigenic cells derived from normal human breast epithelia were classified in to 3 groups according to their characteristics[3, 4].

	CELL LINE	SUBTYPE	ER	PR	HER2
1	184B5	NA	NA	NA	NA
2	AU565	LU	−	−	−
3	BT20	BA	−	−	−
4	BT474	LU	+	+	+
5	BT483	LU	+	+	−
6	BT549	BB	−	−	−
7	CAMA-1	LU	+	−	−
8	DU4475	BA	−	−	NA
9	HCC38	BB	−	−	−
10	HCC70	BA	+	−	−
11	HCC202	NA	−	−	+
12	HCC1187	BA	−	−	−
13	HCC1395	BB	−	−	−
14	HCC1419	LU	−	−	+
15	HCC1428	LU	+	+	−
16	HCC1500	BB	−	−	−
17	HCC1569	BA	−	−	+
18	HCC1599	BA	−	−	−
19	HCC1806	NA	−	−	−
20	HCC1937	BA	−	−	−
21	HCC1954	BA	NA	NA	NA
22	HCC2157	BA	−	+	+
23	HCC2218	LU	−	−	+
24	Hs578T	BB	−	−	−
25	MCF7	LU	+	+	−
26	MCF10A	BB	−	−	−
27	MCF10F	BB	−	−	−
28	MCF12	BB	−	−	−
29	MDAKb2	LU	−	−	−
30	MDA-MB-134-VI	LU	+	−	−
31	MDA-MB-157	BB	−	−	−
32	MDA-MB-175-VII	LU	+	−	−
33	MDA-MB-231	BB	−	−	−
34	MDA-MB-361	LU	+	−	−
35	MDA-MB-415	LU	+	−	−
36	MDA-MB-436	BB	−	−	−
37	MDA-MB-453	LU	−	−	−
38	MDA-MB-468	BA	−	−	−
39	SKBR3	LU	−	−	+
40	UACC812	LU	+	−	−
41	UACC893	LU	−	−	+
42	ZR-75-1	LU	+	−	−
43	ZR-75-30	LU	+	−	+
44	HBL100	BB	+	−	−

The different breast tumor phenotypes were: Basal A (BA); Basal B (BB); and Luminal (LU). NA (not applicable), was used to identify cell subtypes that are not well-characterized. The immunological charateristics of each cell was indicated by either positive (+) or negative (-) for the presence or absence, respectively of the receptors estrogen [ER], progesterone [PR] and human epidermal growth factor receptor 2 (HER2). All cells were acquired and cultured in basic media as recommended by American Type Culture Collection (ATCCA; Manassas, VA USA). The cell characteristics, specifications and history are as reported by ATCCA.

Supplementary Table S2: Primers used for the methylation analysis indicating the direction (forward [FOR] or reverse [REV]) and the first nucleotide position relative to the translation and transcription start sites

Primer	Position	Sequence
FOR	−1762/ −1315	GTTGGGTTGATGTTATGGTTTTTTG
FOR	−1559/ −1112	TGAGTTTGTATTAGGAATAGGTTAGTTAGGTT
FOR	−1554/ −1107	TTGTATTAGGAATAGGTTAGTTAGGTTTAAGG
FOR	−1548/ −1101	TAGGAATAGGTTAGTTAGGTTTAAGGG
FOR	−988/ −541	GATTGTGATTATTTTTTGAAGTTATTAAA
FOR	−949/ −502	GGTAATGAGAAAAGGGAGTGATTTGATG
FOR	−694/ −247	AGTTTGGTAAAGGATTTGGTTTATTTTG
FOR	−616/ −169	AGATAGAAAGAAATTAGGAAGTAAGGGGA
FOR	−613/ −166	AGAAAGAAATTAGGAAGTAAGGGGAAATT
FOR	−600/ −153	GGAAGTAAGGGGAAATTATTATAGTTTG
FOR	−474/ −27	GAATAGATTTGAAATAGGGGGAGAGTT
FOR	−385/ +62	TATTGGGATAAAAGGTAAGATGGTATTA
REV	−1047/ −600	TAAAAACCCAAAATATTACTTTTTCCC
REV	−693/ −246	CAAAATAAACCAAATCCTTTACCAAACT
REV	−616/ −169	TCCCCTTACTTCCTAATTTCTTTCTATCT
REV	−474/ −27	AACTCTCCCCCTATTTCAAATCTATTC
REV	−385/ +62	TAATACCATCTTACCTTTTATCCCAATA
REV	−101/ +346	CACCAAACAACTTTAAAATCAACACC
REV	+17/ +464	AAATAAAATTCTAAATACTCCCAAAACCTCT

All primer sequences are from 5′ to 3′.

Supplementary Table S3: Primers used to amplify the FRK promoter region from genomic DNA and clone the amplicon into the pGL3 reporter plasmid, with the direction and position from the transcriptional start site indicated

Primer	Position	RE	Sequence
FOR	−2832	Kpn1	AACTGAAACATGGTACCCTTCAACCAACATCTCCC
FOR	−2008	Kpn1	GGAGAATTCTGGTACCATGGACACAAAGAAACCTTCTCTC
REV	−380	NheI	TACCCCTCCCATCTCCCACACCC
FOR	−436	Kpn1	GACAAGAAAGGTACCTAAATACTTTAATTTGCCTTTTTAAATTTCCCC
FOR	−308	Kpn1	CAAGACAAAAAGCGGTACCTGGGCTAGCCTGTTTCTCCAGGATGAAAC
FOR	−184	Kpn1	GGTAAAGAGGTCAGAAGGTACCATTTAGATCACAGACAGAAAGAAACC
FOR	−81	Kpn1	CAGCCTCTGCTGGTACCTATTCATTGGCAAAAAGGAGAGAGCCGTCC
FOR	+89	Kpn1	AAAAGGCAAGATGGTACCATTCTGTTCTCAGATATTTGTC
FOR	+156	Kpn1	TATTTTATTTTTGTTGTGGGTACCTTAAGCAGATAAGAAGAAAAGACA
REV	+197	HindIII	TTAGTTAAGCTACTTAGATCGCAGATCTGCTGCTCACTAGGAAGGTGTC

The restriction enzyme (RE) site underlined. All primer sequences are 5′ to 3′. (FOR = Forward; REV = Reverse).

Supplementary Table S4: Primers that were used to introduce the internal deletions within the FRK promoter-pGL3 reporter plasmid

Primer	Position	Sequence
−−74 FOR	−380/ −306	GGTGGCAAAACAATTCTACT**GCTAGC**TGTCAGGGAAAAAAATCTCTG
−74 REV	−380/ −306	CAGAGATTTTTTTCCCTGACA**GCTAGC**AGTAGAATTGTTTTGCCACC
−182 FOR	−299/ −117	GGGGAAATTATTACAGTCTGTT**GCTAGC**TCAGAATGTTAAAGGC
−182 REV	−299/ −117	GCCTTTAACATTCTGA**GCTAGC**AACAGACTGTAATAATTTCCCC
−235 FOR	−299/ −70	GCCTCTGCTGGTTGGTTATTC**GCTAGC**AAAAAGGAGAGAGCCGT
−235 REV	−299/ −70	ACGGCTCTCTCCTTTTT**GCTAGC**GAATAACCAACCAGCAGAGGC
−495 FOR	−299/ +194	AGAAAAGACACCTTCCTA**GCTAGC**AGCTGCCCAGCTCCTGCTCA
−495 REV	−299/ +194	TGAGCAGGAGCTGGGCAGCT**GCTAGC**TAGGAAGGTGTCTTTTCT

The primer direction and positions of the nucleotides deleted from the first to last relative to the transcriptional start site are indicated. The restriction enzyme site (NheI) is in bold and underlined.All primer sequences are 5′ to 3′.(FOR = Forward; REV = Reverse).

Supplementary Table S5: Primers that were used to introduce site mutations into the FRK promoter constructs with the mutation indicated in bold and underlined

Primer	Sequence
M4 FOR	AAAAAA**<u>GCT</u>**TCTGCCGTGAATTTCAGTGCTGGGAACATAACAAGAC
M4 REV	GTCTTGTTATGTTCCCAGCACTGAAATTCACGGCAGA**<u>AGC</u>**
M5 FOR	TATTTGTCAGGGAAAAAAATCTC**<u>ACG</u>**CGTGAATTTCAGTGCTGGGAACA
M5 REV	TGTTCCCAGCACTGAAATTCACG**<u>CGT</u>**GAGATTTTTTCCCTGACAAATA
M6 FOR	CTCTGCCG**<u>CAG</u>**ATTTCAGTGCTGGGAACATAACAAGACAAAAAGCTACC
M6 REV	GGTAGCTTTTTGTCTTGTTATGTTCCCAGCACTGAAAT**<u>CTG</u>**
*M7 FOR	GAAAAAAATCTCTGCCGTGAAT**<u>CCT</u>**AGTGCTGGGAACATAACAAGAC
*M7 REV	GTCTTGTTATGTTCCCAGCACT**<u>AGG</u>**ATTCACGGCAGAGATTTTTTTC
*M8 FOR	GAAAAAAATCTCTGCCGTGAATTTCAGTGCT**<u>AAA</u>**AACATAACAAGAC
*M8 REV	GTCTTGTTATGTT**<u>TTT</u>**AGCACTGAAATTCACGGCAGAGATTTTTTTC
M9 FOR	GA**<u>CCC</u>**TAGCGGCTGCTTTAACAGTTTGGTAAAGGATCTGG
M9 REV	CTGTTAAAGCAGCCGCTA**<u>GGG</u>**TCTATATTCCTTTTGTTTC
M10 FOR	TA**<u>ATA</u>**GCTGCTTTAACAGTTTGGTAAAGGATCTGGTCTAT
M10 REV	ATCTCCCACACCCAAGATAGACCAGATCCTT

All primer sequences are 5′ to 3′. *M7-FOR= STAT-M1; *M7-REV= STAT-M1; *M8-FOR = STAT-M2;*M8-REV= STAT-M2.

Supplementary Table S6: Primers that were used for Chromatin Immuno-Precipitation (ChIP) of the FRK proximal promoter region -462/ -271, from the transcriptional start site (TSS)

Primer	Position from the TSS/+1	Sequence
Forward	−462	TTGCTCTGTGTCTGACAAGAAATACCTAAATACT
Reverse	−271	TTTTGTTTCATCCTGGAGAAACAGGCTAGCCCA

The primer direction and position are indicated from the transcriptional start site (TSS).

Supplementary Table S7: List of the definitions of the acronyms used within the text

ACRONYMS	
ANKRD11	Ankyrin repeat domain 11
ANOVA	One-way analysis of variance
BA	Basal B
BB	Basal B
BRK	Breast tumor kinase
BRCA	breast cancer susceptibility protein
CA-STAT 5	Constitutively active Signal transducer and activator of transcription 5
CDKN1A	Cyclin dependent kinase inhibitor 1A
CDKN2A	Cyclin dependent kinase inhibitor 2A
CpG	5'- Cytsoine - phosphate - Guanine -3'
DAC	Decitabinetreatment
DMSO	Dimethyl Sulfoxide
E2F	E2F transcription factor
FOG	Friend of GATA
FRK	Fyn-related kinase
GAPDH	Glyceraldehyde 3-phosphate dehydrogenase
GFI1	Growth factor independence 1
HDAC	Histone deacetylase
HDI	Histone deacetylase inhibitors
H3K9me3	Histone H3 lysine 9 trimethylation
H3K27me3	Histone H3 lysine 27 trimethylation
H4 acetylation	Histone H4 acetylation
HNF6	Hepatocyte nuclear factor 6
LU	Luminal
**ACRONYMS**	
MAP	Mitogen Activated Protein
PAX_Q6	Paired box genes
pRB	Retinoblastoma protein
PTEN	Phosphatase and Tensin homologue deleted on chromosome 10
P73/ TP73	Tumor protein 73
RAR	Retinoic acid receptor
RARb2	Retinoic acid receptor beta2
RPL13A	Ribosomal Protein L13A
SALL3	Spalt like transcription factor 3
SFK	Src family kinases
SH	Src homology
SRMS	Src-Related Kinase Lacking C-Terminal Regulatory Tyrosine And N-Terminal Myristylation Sites
TERT	telomerase reverse transcriptase
TSA	Trichostatin A
TSS	Transcriptional start site /+1
TST1/ POU3	Teste-1/ POU class 3 homeobox 1
UCSC	University of California Santa Cruz
5′ UTR	5′ un-translated region

Supplementary Table S8: Acronyms used to define the tumor types as adopted from (Huang, Hsu et al. 2015)

ACRONYMS	DIFFERENT TUMOR TYPES
BLCA	Bladder Urothelial Carcinoma
BRCA	Breast Invasive Carcinoma
CESC	Cervical Squamous Cell Carcinoma and Endocervical Adenocarcinoma
COAD	Colon Adenocarcinoma
HNSC	Head and Neck Squamous Cell Carcinoma
KIRC	Kidney Renal Clear Cell Carcinoma
KIRP	Kidney Renal Papillary Cell Carcinoma
LIHC	Liver Hepatocellular Carcinoma
LUAD	Lung Adenocarcinoma
LUSC	Lung Squamous Cell Carcinoma
PAAD	Pancreatic Adenocarcinoma
PRSD	Prostate Adenocarcinoma
READ	Rectum Adenocarcinoma
SARC	Sarcoma
SKCM	Skin Cutaneous Melanoma
STAD	Stomach Adenocarcinoma
THCA	Thyroid Carcinoma
UCEC	Uterine corpus Endometrial Carcinoma

Huang, W. Y., S. D. Hsu, et al. (2015). "MethHC: a database of DNA methylation and gene expression in human cancer." Nucleic Acids Res 43(Database issue): D856-861.
